# Gut microbiota reflect adaptation of cave-dwelling tadpoles to resource scarcity

**DOI:** 10.1093/ismejo/wrad009

**Published:** 2024-01-10

**Authors:** Wei Zhu, Liming Chang, Shengchao Shi, Ningning Lu, Simeng Du, Jiatang Li, Jianping Jiang, Bin Wang

**Affiliations:** Chinese Academy of Sciences Key Laboratory of Mountain Ecological Restoration and Bioresource Utilization & Ecological Restoration Biodiversity Conservation Key Laboratory of Sichuan Province, Chengdu Institute of Biology, Chinese Academy of Sciences, Chengdu 610041, Sichuan, China; Chinese Academy of Sciences Key Laboratory of Mountain Ecological Restoration and Bioresource Utilization & Ecological Restoration Biodiversity Conservation Key Laboratory of Sichuan Province, Chengdu Institute of Biology, Chinese Academy of Sciences, Chengdu 610041, Sichuan, China; Chinese Academy of Sciences Key Laboratory of Mountain Ecological Restoration and Bioresource Utilization & Ecological Restoration Biodiversity Conservation Key Laboratory of Sichuan Province, Chengdu Institute of Biology, Chinese Academy of Sciences, Chengdu 610041, Sichuan, China; Chinese Academy of Sciences Key Laboratory of Mountain Ecological Restoration and Bioresource Utilization & Ecological Restoration Biodiversity Conservation Key Laboratory of Sichuan Province, Chengdu Institute of Biology, Chinese Academy of Sciences, Chengdu 610041, Sichuan, China; Chinese Academy of Sciences Key Laboratory of Mountain Ecological Restoration and Bioresource Utilization & Ecological Restoration Biodiversity Conservation Key Laboratory of Sichuan Province, Chengdu Institute of Biology, Chinese Academy of Sciences, Chengdu 610041, Sichuan, China; Chinese Academy of Sciences Key Laboratory of Mountain Ecological Restoration and Bioresource Utilization & Ecological Restoration Biodiversity Conservation Key Laboratory of Sichuan Province, Chengdu Institute of Biology, Chinese Academy of Sciences, Chengdu 610041, Sichuan, China; Chinese Academy of Sciences Key Laboratory of Mountain Ecological Restoration and Bioresource Utilization & Ecological Restoration Biodiversity Conservation Key Laboratory of Sichuan Province, Chengdu Institute of Biology, Chinese Academy of Sciences, Chengdu 610041, Sichuan, China; Chinese Academy of Sciences Key Laboratory of Mountain Ecological Restoration and Bioresource Utilization & Ecological Restoration Biodiversity Conservation Key Laboratory of Sichuan Province, Chengdu Institute of Biology, Chinese Academy of Sciences, Chengdu 610041, Sichuan, China

**Keywords:** cave adaptation, glycosidase, metagenome, metamorphosis, plasticity

## Abstract

Gut microbiota are significant to the host’s nutrition and provide a flexible way for the host to adapt to extreme environments. However, whether gut microbiota help the host to colonize caves, a resource-limited environment, remains unknown. The nonobligate cave frog *Oreolalax rhodostigmatus* completes its metamorphosis within caves for 3–5 years before foraging outside. Their tadpoles are occasionally removed from the caves by floods and utilize outside resources, providing a contrast to the cave-dwelling population. For both cave and outside tadpoles, the development-related reduction in their growth rate and gut length during prometamorphosis coincided with a shift in their gut microbiota, which was characterized by decreased *Lactobacillus* and *Cellulosilyticum* and *Proteocatella* in the cave and outside individuals, respectively. The proportion of these three genera was significantly higher in the gut microbiota of cave-dwelling individuals compared with those outside. The cave-dwellers’ gut microbiota harbored more abundant fibrolytic, glycolytic, and fermentative enzymes and yielded more short-chain fatty acids, potentially benefitting the host’s nutrition. Experimentally depriving the animals of food resulted in gut atrophy for the individuals collected outside the cave, but not for those from inside the cave. Imitating food scarcity reproduced some major microbial features (e.g. abundant *Proteocatella* and fermentative genes) of the field-collected cave individuals, indicating an association between the cave-associated gut microbiota and resource scarcity. Overall, the gut microbiota may reflect the adaptation of *O. rhodostigmatus* tadpoles to resource-limited environments. This extends our understanding of the role of gut microbiota in the adaptation of animals to extreme environments.

## Introduction

Cave-dwelling is an extreme phenomenon that reveals some mechanisms of physical and functional adaptation to challenging environments. Resource scarcity is a major characteristic of cave environments and it exerts strong selective pressures on the colonizing species [1]. Obligate cave vertebrates, such as cavefish (e.g. members of *Amblyopsidae*, *Bythitidae*, *Sinocyclocheilus*, and *Astyanax*) [2-4] and cave salamanders (e.g. members of *Eurycea* and *Speleomantes*) [5, 6], spend their entire life in resource-limited environments. Consequently, they have evolved remarkable physiological and metabolic adaptations (e.g. improved assimilation efficiency, a reduced metabolic rate, and increased fat storage) through genetic mutations [7-10]. By contrast, some anuran species exhibit a nonobligate cave-dwelling life cycle (i.e. external foraging but internal (cave) egg laying), where tadpoles develop and metamorphose with a typical cave-adapted morphology (i.e. degenerated eyes and transparent skin; Supplementary Fig. S1A–C) [11-13]. The different requirements of cave and outside lifestyles may limit the path to genetic adaptation [13, 14]. For instance, the constitutive physiological and metabolic changes that enhance tolerance to starvation in cave-based life stages (e.g. a reduced metabolic rate) may not suit the external life stages, as the cave frogs experience resource scarcity only in their larval stages. Therefore, cave frogs may adopt more flexible adaptive strategies, which are likely to be distinct from those of obligate cave dwellers, to cope with resource scarcity.

Gut microbiota are involved in the host’s behavior, health, immunity, nutrition, and metabolism [15-18]. The host and its commensal microbiome can act as a unit and undergo evolutionary selection [19]. Unlike the highly conserved genome of the host, the microbial genome is highly plastic and can change rapidly in response to environmental variations [20-22]. In some cases, environment-adapted symbiotic microbiomes are essential for animals to colonize new environments and cope with lifestyle transitions [23, 24]. Gut microbes have been reported to be closely associated with the nutritional strategy of the animal hosts [25-29]. The genetic material of gut microbes can complement the host’s genome in digestive and biosynthetic functions [30]. For example, cellulose is a major dietary component in ruminants, but it cannot be catabolized by their digestive systems. Instead, cellulolytic symbionts found in their digestive tracts can break down and convert cellulose to its fermented end products, short-chain fatty acids (SCFAs) [31], which can be used as metabolic substrates by the host [32, 33]. Moreover, the community structure of symbiotic microbiota can be highly plastic in response to changes in external factors, including the composition and availability of food [34-37]. This plasticity allows the dynamic reorganization of the gut microbiota according to the environment and thus maintains the host’s metabolic homeostasis by modulating the efficiency of nutritional intake and the rate of energy consumption [23, 37-39]. Therefore, we hypothesize that the gut microbiomes and metagenomes may be associated with the adaptation of frogs to cave environments.

*Oreolalax rhodostigmatus* (Megophryidae, Anura) is a nonobligate cave frog species that inhabits the karst caves in southwestern China [40]. They develop in shallow cave pools that originate from subterranean ravine streams (Supplementary Fig. S1A and B). Most *O. rhodostigmatus* tadpole populations are obligate cave-dwellers characterized by degenerated eyes and transparent skin (Supplementary Fig. S1C), while the black-skinned adults have well-developed eyes and can forage outside the cave (Supplementary Fig. S1D) [41, 42]. The tadpoles require 3–5 years to complete their aquatic life stage in the caves. Despite the limited resources in the cave, the *O. rhodostigmatus* tadpoles can grow up to 12 cm in length. To examine whether the gut microbiota of *O. rhodostigmatus* tadpoles exhibit some metabolic functions that potentially benefit the host in a resource-limited environment, we conducted three comparative experiments and tested three hypotheses.

The first hypothesis we tested is whether changes in the host’s growth status are associated with shifts in its gut microbiota during metamorphosis. Amphibian tadpoles exhibit a high degree of developmental plasticity that enables them to decouple growth (premetamorphosis and prometamorphosis, before Gosner Stage 36 and Stages 36–41, respectively) and differentiation (metamorphic climax, Gosner Stages 42–45) to a remarkable degree [43, 44]. During metamorphic climax, a nonfeeding stage for most amphibian species, the animals partly use the body mass accumulated in the previous stages as metabolic substrates [45]. The tadpoles usually reach their maximum body size and stop growing at the prometamorphic stages [43, 46, 47], which means a reduced demand for environmental resources. Thus, we hypothesized that the development-related changes in the host’s growth status and nutrient-related traits at the prometamorphic stages would be accompanied by a significant shift in gut microbiota.

**Figure 1:**
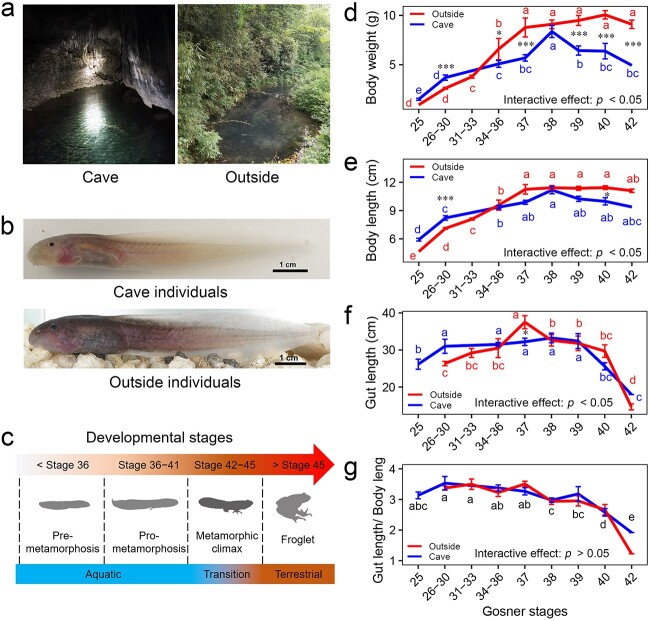
Difference in living environments, morphology, and physiology between cave and outside tadpoles; (A–B) typical microhabitats and morphology of *O. rhodostigmatus* tadpoles; (C) developmental stages of *O. rhodostigmatus* tadpoles; (D–G) changes in tadpoles’ body weight, body length, gut length, and relative gut length (gut length/body length) across environments and developmental stages; note that the gut length also decreased drastically from Gosner Stages 40–42; this was due to the onset of metamorphic climax at Stage 42; different letters in panel D–F indicate significant differences between stages for cave or outside individuals (simple effect analysis for ANOVA, with significant interactive effect); different letters in panel G indicate significant differences between stages for all individuals (two-way ANOVA followed by the S-N-K post-hoc test, without a significant interactive effect); asterisks indicate differences between cave and outside individuals at specific stages (simple effect analysis for ANOVA, with significant interactive effects): ^*^P < .05; ^*^^*^P < .01; ^*^^*^^*^P < .001.

The second hypothesis was based on a comparative analysis of the gut microbial composition and function between *O. rhodostigmatus* individuals living inside and outside the caves (hereafter referred to as cave and outside individuals). Generally, *O. rhodostigmatus* tadpoles cannot leave the caves during their aquatic life stages. However, we unexpectedly found a population of *O. rhodostigmatus* tadpoles inhabiting and foraging in a shallow pool outside the cave (Fig. 1A and B). This provided us with an opportunity to study the flexibility of gut microbiota in response to resource availability. We hypothesized that the gut microbiota of cave individuals (or cave-associated gut microbiota) would feature microbes and metabolic functions that potentially benefit the host in a resource-limited environment (e.g. unconventional resource degradation and metabolic capacity).

For the third hypothesis, we performed a laboratory experiment to investigate how the food availability affected the physiological status and gut microbiota of *O. rhodostigmatus* tadpoles from the cave and outside habitats (hereafter referred to as cave- and outside-derived individuals). We hypothesized that the cave- and outside-derived individuals would differ in some physiological traits related to nutrition in response to starvation, and we expected that low food levels would reproduce the major microbial features (i.e. microbial composition and function) of field-collected cave individuals.

## Materials and methods

### Animal collection

We collected *O. rhodostigmatus* tadpoles from a stream in a karst cave and outside (downstream) in Tongzi County, Guizhou Province, China (28.50 N, 107.05 E; Supplementary Fig. S1E) in 2019 and 2020. The water of the stream in the cave had a relatively stable temperature of ~15°C. We chose this cave for two reasons: (i) it had a large population and (ii) it had both cave and outside populations. The tadpoles within the cave were collected from multiple pools. These pools are connected by the stream flowing through the cave. Environmental samples for chemical and microbial analyses were also taken from several pools. All the outside tadpoles were collected from the same pool downstream from the cave pools. The outside individuals were probably removed from the inner caves by flooding, as the cave and outside pools were also connected by the stream (see more information in the caption of Supplementary Fig. S1). Despite their different appearance (Fig. 1B), the cave and outside tadpoles shared common genetic background, as indicated by their placements within nested branches of the phylogenetic tree (Supplementary Fig. S2; see the methods used for the phylogenetic analyses in Supplementary Data 1). We measured water pH, oxidation reduction potential (ORP), and chlorophyll levels in the cave and outside waters with a Manta+3.5 water quality monitor (*n* = 8 for each group) (Eureka, USA). The water bodies they inhabited were ecologically distinct (Fig. 1A), with a higher pH, a lower ORP, and a lower chlorophyll level (a major index of water nutrition level) detected in the cave water body (Supplementary Fig. S1F). The developmental stages of tadpoles were identified by following the criteria of Gosner [43]. Tadpoles in this study were at their Stages 25–42 (see the detailed description on the identification of the stages in Supplementary Data 1). Considering that the digestive tract would undergo reorganization during the metamorphic climax (Stages 42–45), we only analyzed the gut microbiota of tadpoles at their premetamorphic (Stages <36) and prometamorphic (Stages 36–41) stages (Fig. 1C). The tadpoles were also measured for body weight, body length, gut length, and eye diameter (see raw data in Supplementary Data 2: Supplementary Tables S1 and S2). After morphological measurements, the tadpoles were euthanized by MS-222 and dissected for tissue collection. All animal procedures were performed according to the protocols approved by the Animal Care Advisory Committee of the Chengdu Institute of Biology, Chinese Academy of Sciences, China (permit number: CIB20191084).

### Food gradient treatment

We collected cave and outside tadpoles at Gosner Stages 25–30. We randomly divided tadpoles with similar body sizes into eight plastic tanks (29 × 18 × 13 cm, with 2 l of water). Each tank contained six cave and five outside individuals, ensuring that each tank had the entire set of cave and outside gut microbes, allowing for the free reorganization of the gut microbiota. We randomly assigned the eight tanks to four groups: the low food level group (L), the middle food level group (M), the high food level group (H), and the very high food level group (VH). Each group included two replicates. We placed the tanks in an artificial climate chamber (RDN-260B, Yanghui, China) set to 15.5°C (D:L = 24:0). We fed the tadpoles with spirulina powder (China National Salt Industry Corporation), for which the nutrient composition has been reported previously [48]. The L, M, H, and VH groups received, respectively, 3, 15, 75, and 225 mg of spirulina powder twice per day. The treatment lasted for 30 days, and we replaced the water every 2 days. We estimated the food gradient according to our experiences in tadpole breeding [48, 49]. We evaluated the validity of this gradient by measuring the tadpoles’ energy status (fat body weight and liver size) and gall-bladder morphology at the end of the experiment. Enlarged dark-green gall bladders and small livers indicated starvation in the tadpoles, while fatty livers and light-colored gall bladders suggested good nutrition [48]. After taking the morphological measurements, we euthanized the tadpoles with MS-222 and dissected them for tissue collection. Note that we could distinguish the cave- and outside-derived individuals from each other according to their skin color.

### 16S ribosomal RNA gene-based microbiome analyses

For the 16S rRNA gene diversity analysis of the gut microbiota, each sample contained the whole gut content of one tadpole (Stages 25–40; see the sample sizes in Supplementary Table S3). We extracted DNA from the samples using a QIAamp DNA Stool minikit (Qiagen, Valencia, CA). We amplified the entire region of the 16S rRNA gene with the primers 27F (AGRGTTTGATYNTGGCTCAG) and 1492R (TASGGHTACCTTGTTASGACTT) (detailed in Supplementary Data 1). After PCR amplification and product purification, we performed high-throughput sequencing using a PacBio platform from Mingke Biotechnology Co., Ltd (Hangzhou, China). The circular consensus sequences (CCSs) were filtered using lima v1.7.0, and the primers were removed using Cutadapt 1.9.1 [50]. After removing chimeras with UCHIME 8.0 [51], the QIIME 2 (version 2020.6) pipeline [52] was used to process the CCSs, and ASVs were obtained after denoising with DADA2 [53]. Annotation was conducted by querying against SILVA v138 [54]. The absolute abundance was normalized using a standard sequence number (the fewest sequences among samples). The alpha-diversity indices and beta-diversity matrices were calculated with the QIIME 2 pipeline.

For 16S rRNA gene diversity analysis of the environmental microbiota, each sample consisted of water sediment from one pool (six samples per group). We amplified the V4–V5 region with the primers 515F (GTGCCAGCMGCCGCGGTAA) and 907R (CCGTCAATTCCTTTGAGTTT) (see the detailed parameters in Supplementary Data 1). After product purification, we performed high-throughput sequencing of the amplicons using a NovaSeq 6000 System (Illumina, PE250) from Mingke Biotechnology Co., Ltd (Hangzhou, China). The raw data were filtered using Trimmomatic 0.33 [55], and the primers were removed using Cutadapt 1.9.1 [50]. Then, USERACH 10 was used to assemble the reads [56], and UCHIME 8.0 was used to remove any chimeras [51]. The QIIME 2 (version 2020.6) pipeline [52] was used to process the sequences, and ASVs were obtained after denoising with DADA2 [53]. The subsequent analyses are identical to those for the tadpole gut microbiota.

### Metagenomic sequencing and data analyses

We sequenced the gut metagenomes of the cave and outside individuals at Stages 26–30 (four samples per group), as well those of individuals from the laboratory experiment (three samples per group). For each sample, which was pooled from the whole gut content of two to four tadpoles, 1 μg of genomic DNA was used for paired-end (PE) library preparation with Illumina’s TruSeq. The libraries were sequenced at Mingke Biotechnology Co., Ltd (Hangzhou, China) using a HiSeq 4000 System (Illumina, PE 150). The HiSeq reads were filtered with custom Perl scripts and Trimmomatic (parameters: Trimmomatic-0.30.jar PE-phred33 LEADING:0 TRAILING:20 SLIDINGWINDOW:50:20 MINLEN:50) to remove low-quality reads [55]. The remaining reads were queried against the *Leptobrachium leishanense* genome (genetically close to *O. rhodostigmatus*) to remove potential host contaminants (threshold identity >97%). Metagenomic assembly and gene prediction were conducted using Megahit and Prodigal, respectively [57, 58]. The predicted genes were queried against the Non-Redundant Protein Sequence (NR) database to obtain the putative taxon assignments of these genes per metagenome [59]. Reads mapped to the host or diet (contamination genes) were removed. A new round of assembly, gene prediction, and annotation was conducted with the retained reads. CD-HIT soft ware was used to construct nonredundant gene sets with <90% overlap and <95% shared sequence identity from the gene files [60]. Salmon was used to determine the relative abundance (transcripts per million (TPM) reads) of the nonredundant gene profiles in each metagenome [61]. Finally, the clean nonredundant gene sequences were queried against the Kyoto Encyclopedia of Genes and Genomes (KEGG) database (threshold e-value: <1e-5; identity: >30) and carbohydrate-active enzymes (CAZymes) databases (threshold e-value <1e-5). The KEGG Orthology entries and pathways, Enzyme Commission numbers, and CAZyme categories associated with each sequence were determined. We calculated the relative abundance of the KEGG pathways and CAZyme categories.

### Measurement of short-chain fatty acids

For measurement of the SCFAs, each sample contained the whole gut content of one tadpole (Stages 30–36), and eight samples were prepared for each group. For each sample, we ground and homogenized 50 mg of the gut contents in a tube with 50 μl of 15% phosphoric acid, 100 μl of water (with 125 μg/ml 4-methylvaleric acid as an internal standard), and 400 μl of ethyl ether for 1 min. We centrifuged the mixture for 10 min (12 000 rpm, 4°C) and collected the supernatant for gas chromatography–mass spectrometry (GC–MS) analysis. We performed the chromatography using a Thermo TRACE 1310-ISQ GC–MS (Thermo, USA) equipped with an Agilent HP-INNOWAX column (Agilent Technologies, USA). The parameters and abundance table are detailed in Supplementary Data 1 and 3.

### Metabolic profiling

We performed metabolic profiling on the gut contents of cave and outside individuals (eight samples per group). Each sample contained the entire gut contents of one tadpole (Stages 30–36). For each sample, we ground 100 mg of the gut contents in liquid nitrogen and extracted it with 1 ml of a methanol:acetonitrile:water mixture (2:2:1, v/v). Then we subjected the extract to ultrasonication for two cycles of 30 min each and incubated it at −20°C for 1 h. After centrifuging the extract at 14 000 rpm for 20 min (4°C), we transferred the supernatants to new tubes and added L-glutamate-d5 as the internal standard. We freeze-dried the samples and reconstituted them in 100 μl of acetonitrile:water (1:1, v/v) for analysis. We used a UPLC-MS system (Nexera X2 LC-30 AD, Shimadzu, Japan; QTRAP 5500, AB SCIEX, USA) with an ACQUITY UPLC BEH Amide column (1.7 μm, 2.1 mm × 100 mm, Waters, USA) for chromatography (see the detailed protocol in Supplementary Data 1). We used MultiQuant v3.3 (AB SCIEX, USA) to extract the peak areas and retention times. We identified the metabolites by comparing their retention times and molecular weights with chemical standards (Supplementary Data 4).

### Statistical analysis

We used IBM SPSS v21.0 for basic statistical analyses. We checked the normality of the data with the Kolmogorov–Smirnov and Shapiro–Wilk tests. We used two-way analysis of variance (ANOVA) to analyze the effects of tadpoles’ source (i.e. cave and outside) and development stages (or food levels) on their body traits, microbial alpha-diversity, and relative abundance. If there were significant interactive effects in the ANOVA models, simple effects tests were conducted for pairwise comparison; if not, least significant difference (LSD) *post hoc* tests were performed for pairwise comparisons of development stages or food levels. We used the linear discriminant analysis effect size (LEfSe) for microbial differential analysis (the Galaxy platform, http://huttenhower.sph.harvard.edu/galaxy/), with a threshold of *P* < .01 and a linear discrimination analysis (LDA) score of >2 or 4 [62]. We used principal-coordinate analysis (PCoA) and permutational multivariate analysis of variance (PERMANOVA) to compare the beta-diversity among groups [63]. We used the Mann–Whitney *U*-test for differential analyses of microbial relative abundance. We used Student’s *t*-test for differential analyses on the metagenomic and metabolomic data. Benjamini–Hochberg correction was used to adjust the *P*-values. We used partial least squares regression with the default parameters to screen bacterial groups whose relative abundance correlated with the food levels (SIMCA v13.0).

## Results

### Development-related changes in the physiological status of *O. rhodostigmatus* tadpoles

We found significant interactive effects between the environments and developmental stages on tadpoles’ body weight and length, orbital diameter, and gut length (Figs 1D–G and S3; details in Supplementary Tables S1 and S2). Although the outside individuals had larger body size than the cave individuals after Stages 34–36, their maximum body sizes were comparable (*P* > .05 for Stage 38, simple effects; Fig. 1D and E). The body size of outside individuals grew considerably before Stage 37 and reached a plateau, while that of cave individuals peaked at Stage 38 and then declined significantly (*P* < .05, simple effects; Fig. 1D and E). The orbital diameters of both cave and outside individuals increased significantly with development and reached temporary plateaus at Stages 37 and 38, respectively (*P* < .05, simple effects; Supplementary Fig. S3). These results suggest reduced somatic growth after Stages 37–38.

The length of digestive tract is plastic to the resource type and availability in the environment [64, 65]. Despite the differences in the resource abundance, the cave and outside individuals had different gut lengths only at Stage 37 (*P* < .05, simple effects), and they were not different in their relative gut length (the ratio of gut length to body length) (*P* > .05, two-way ANOVA; Fig. 1F and G). The gut length of outside individuals peaked at Stage 37 and then decreased significantly (*P* < .05, simple effects), while that of cave individuals remained unchanged before Stage 39 and then decreased drastically (*P* < .05, simple effects). These results suggest a physiological transition of *O. rhodostigmatus* tadpoles at the prometamorphic stages.

### Changes in gut microbiota with the growth status of *O. rhodostigmatus* tadpoles

**Figure 2:**
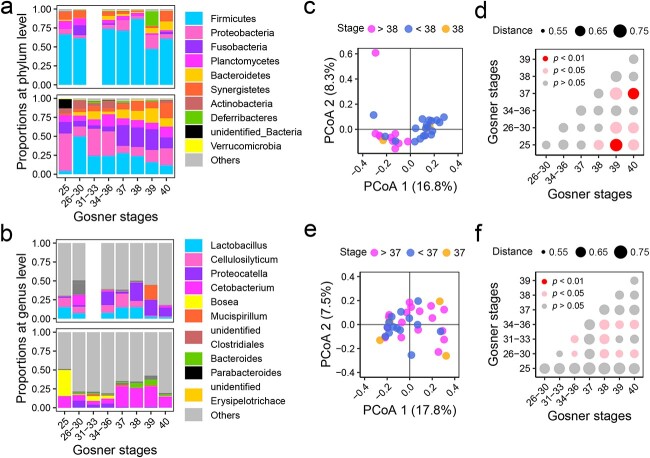
Development-related changes in the gut microbiota of *O. rhodostigmatus* tadpoles; composition of the gut microbiota composition at the phylum (A) and genus (B) levels; upper, cave individuals; lower, outside individuals; (C) PCoA plot showing the development-related changes in the microbial beta-diversity of cave individuals (unweighted UniFrac distances); (D) pairwise PERMANOVA of unweighted UniFrac distances of cave individuals at different stages; (E) PCoA plot showing the development-related changes in the microbial beta-diversity of outside individuals (unweighted UniFrac distances); (F) pairwise PERMANOVA of unweighted UniFrac distances of outside individuals at different stages.

The hosts’ physiological changes were accompanied by a significant shift in the microbial community structure for both cave and outside individuals. The gut microbiota of cave individuals were dominated by *Firmicutes* (67.1%), while that of outside individuals were mainly composed of *Firmicutes* (24.2%), *Proteobacteria* (20.0%), and *Fusobacteria* (18.0%) (Fig. 2A). Bacterial genera with a relative abundance of over 5% included *Proteocatella* (11.0%), *Lactobacillus* (9.8%), and *Cellulosilyticum* (8.7%) in cave individuals, and *Cetobacterium* (18.0%) in outside individuals (Fig. 2B). The cave individuals tended to have a significantly different gut microbiota before and after Stage 38 (*P* < .05, PERMANOVA; Fig. 2C and D). Similarly, the outside individuals tended to have a significantly different gut microbiota before and after Stage 37 (*P* < .05, PERMANOVA; Fig. 2E and F). Specifically, the gut microbiota of cave individuals showed increased *Bacteroidales* and decreased *Lactobacillus* and *Cellulosilyticum*, two predominant genera, after Stage 38 (*P* < .01 and LDA > 4, LEfSe; Supplementary Fig. S4A and B). The gut microbiota of outside individuals showed decreased *Proteocatella*, *Microbacteriaceae*, *Rhizobiaceae*, *Bosea*, and *Methylobacterium* after Stage 37 (*P* < .01 and LDA > 4, LEfSe; Supplementary Fig. S4C and D); among these, *Proteocatella* was notable for its high relative abundance (6.2%) in individuals at Stages 26–36 (Fig. 2B).

### Compositional and functional differences in the gut microbiota between cave and outside tadpoles

**Figure 3:**
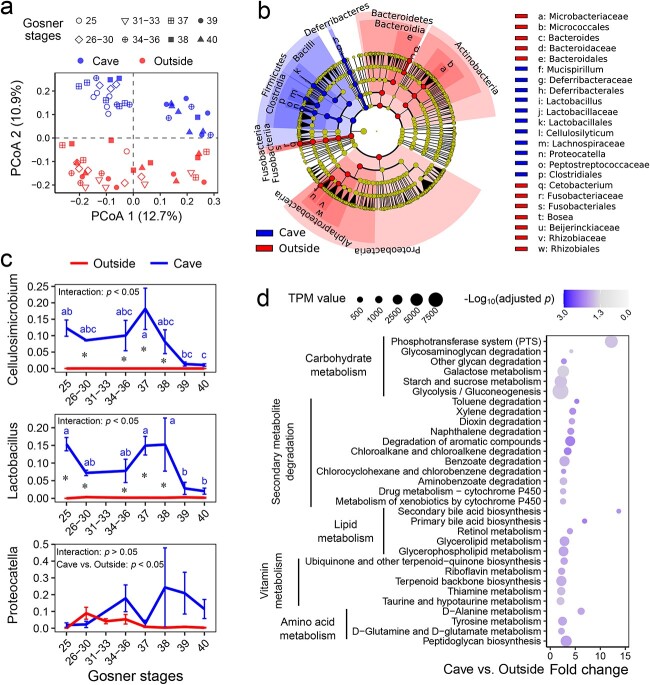
Differences in the microbial composition and metabolic function between cave and outside tadpoles; (A) PCoA plot of unweighted UniFrac distances; (B) differential analyses of the microbiota between cave and outside individuals based on the LEfSe at a threshold of *P* < .01 (Kruskal–Wallis and Wilcoxon tests) and an LDA score of >4; (C) variations in relative abundance of the primary differential genera across developmental stages; the *y*-axis denotes the proportions; different letters indicate significant differences between stages for cave individuals, while asterisks denote significant difference between cave and outside individuals at a given stage (two-way ANOVA and simple effect analysis); (D) differential KEGG metabolic pathways (*P* < .005 and adjusted *P* < .05) between the gut metagenomes of cave and outside tadpoles (based on the relative abundance of metabolic genes).

Cave individuals showed significantly lower alpha-diversity (Shannon and PD_whole_tree indices) in the gut microbiota than outside individuals (*P* < .05, two-way ANOVA; Supplementary Fig. S5A). There were prominent differences in the microbial composition between cave and outside individuals (*P* < .001, PERMANOVA; Fig. 3A and Supplementary Fig. S5B and C). Specifically, cave individuals had a significantly higher relative abundance of *Firmicutes*, *Deferribacteres*, *Clostridia*, *Bacilli*, *Proteocatella*, *Lactobacillus*, and *Cellulosilyticum*, while outside individuals had a higher relative abundance of *Proteobacteria*, *Actinobacteria*, *Bacteroidetes*, *Fusobacteria*, *Cetobacterium*, *Bosea*, and *Bacteroides* (*P* < .01 and LDA > 4, LEfSe; Fig. 3B). The relative abundance of *Proteocatella*, *Lactobacillus*, and *Cellulosilyticum*, which exhibited changes coinciding with the hosts’ growth status, accounted for the major microbial differences between the cave and outside groups (Fig. 3C). The differences between cave and outside individuals were unlikely to have been caused by the microbial differences in the environment. The predominant bacterial genera in the gut were almost absent in the environment (Supplementary Fig. S6A–E). Only nine bacterial genera varied in their relative abundance between the cave and the outside in both the gut and the environment, and seven of them showed opposite trends in the gut and the environment (Supplementary Fig. S6F and G). The functions of the gut microbiota were also different between cave and outside individuals (*P* < .001, PERMANOVA; Supplementary Fig. S7A and B). The majority of the differential metabolic genes (adjusted *P* < .05) exhibited a higher relative abundance in the gut metagenome of cave individuals (Supplementary Fig. S7C), especially genes involved in carbohydrate metabolism (e.g. carbohydrate absorption and degradation), lipid metabolism (e.g. bile acid biosynthesis), secondary metabolite degradation (e.g. aromatic component degradation), vitamin metabolism, and amino acid metabolism (Fig. 3D).

**Figure 4:**
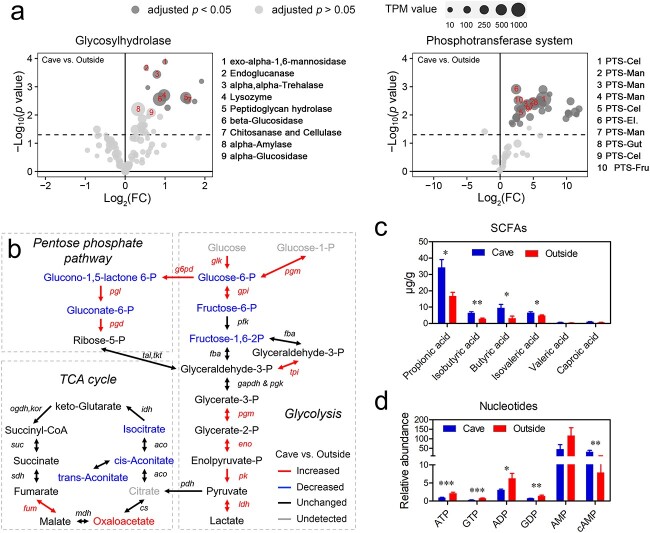
Comparative analysis of the gut metagenome and metabolome; the gut metagenome and metabolome were measured in tadpoles at Stage 26–30 (*n* = 4 per group) and Stages 30–36 (*n* = 8 per group), respectively; (A) volcano plots presenting the variations in the relative abundance of genes involved in GH and sugar PTS components; the horizontal axis denotes the fold change (FC) in TPM, and the vertical axis gives the *P*-values; the size of the dots denotes the maximum TPM value across samples; (B) network representing the metabolic differences between cave and outside microbiota; (C-D) relative abundance of SCFAs (C) and nucleotides (D) in the tadpoles’ gut contents; the values show the mean ± SE; ^*^ adjusted *P* < .05; ^*^^*^adjusted *P* < .01; ^*^^*^^*^adjusted *P* < .001 (Student’s *t*-test and BH correction).

Metagenomic and metabolomic analyses were combined to analyze the differences in carbohydrate catabolism between cave and outside gut microbiota (Fig. 4). Most of the differential CAZymes showed a higher relative abundance in the cave microbiota (adjusted *P* < .05; Supplementary Fig. S7D), especially the glycosyl hydrolases (GHs) (Supplementary Fig. S7E). These included GH8 (chitosanase and cellulase activity), GH1 (β-glucosidase activity), and GH13 (α-glucosidase), which exhibited high relative abundance or a remarkable fold change (Fig. 4A). The gut metagenomes of cave individuals were also richer in sugar phosphotransferase system (PTS) components, especially those transporting cellobiose, mannose, glucose, fructose, and sucrose (adjusted *P* < .05; Fig. 4A). After that, the carbohydrate metabolic flow comes to glycolysis. The gut metagenome of cave individuals had a higher relative abundance of glycolytic genes (e.g. glucose-6-phosphate isomerase/*gpi*, 1-phosphofructokinase/*fruk*, and *enolase*) (adjusted *P* < .05; Supplementary Fig. S7F). In line with this genetic difference, the relative levels of early phase glycolytic metabolites (e.g. glucose 6-phosphate, fructose 6-phosphate, and fructose 1,6-biphosphate) were lower in the gut content of cave individuals, but their late-phase glycolytic metabolites (e.g. glycerate 3-phosphate, pyruvate, and lactate) reached similar levels to that of their outside counterparts (at a threshold of adjusted *P* < .05; Fig. 4B). This implies enhanced glycolysis in the gut microbiota of the cave individuals compared with their outside counterparts. The metabolic flux from glycolysis can either enter the tricarboxylic acid cycle (TCA) cycle for aerobic metabolism or it can undergo fermentation. The relative abundance of microbial TCA cycle genes was not higher in cave individuals compared with those from outside (Supplementary Fig. S7G). Cave individuals had relatively more oxaloacetate and less *cis*-aconitate, *trans*-aconitate, and isocitrate in their gut content than outside individuals (adjusted *P* < .05; Fig. 4B). This pattern of variation implied that the metabolic flux through the TCA cycle was relatively lower in the gut microbiota of cave individuals, as conversion from oxaloacetate to citrate is the first step to divert the flux into the cycle (Fig. 4B). Unlike the TCA cycle, microbial genes for SCFA biosynthesis were overrepresented in the gut of cave individuals (Supplementary Fig. S7H), and higher levels of SCFAs (e.g. propionic acid, butyric acid, and isobutyric acid) were detected in the gut content of cave individuals (adjusted *P* < .05; Fig. 4C). Fermentation is much less efficient for producing adenosine triphosphate (ATP) than aerobic metabolism. The gut content of cave individuals showed lower relative levels of ATP, guanosine triphosphate (GTP), adenosine diphosphate (ADP), and guanosine diphosphate (GDP), but a higher relative level of cyclic adenosine monophosphate (cAMP) (adjusted *P* < .05; Fig. 4D). This was a sign of energy deficiency and suggested that a larger proportion of resources were allocated to fermentation of SCFAs rather than energy production in cave-associated microbiota. Overall, these results imply that the gut microbiota of the cave individuals was fibrolytic and fermentative.

### Food abundance shapes the gut microbiota of *O. rhodostigmatus* tadpoles

**Figure 5:**
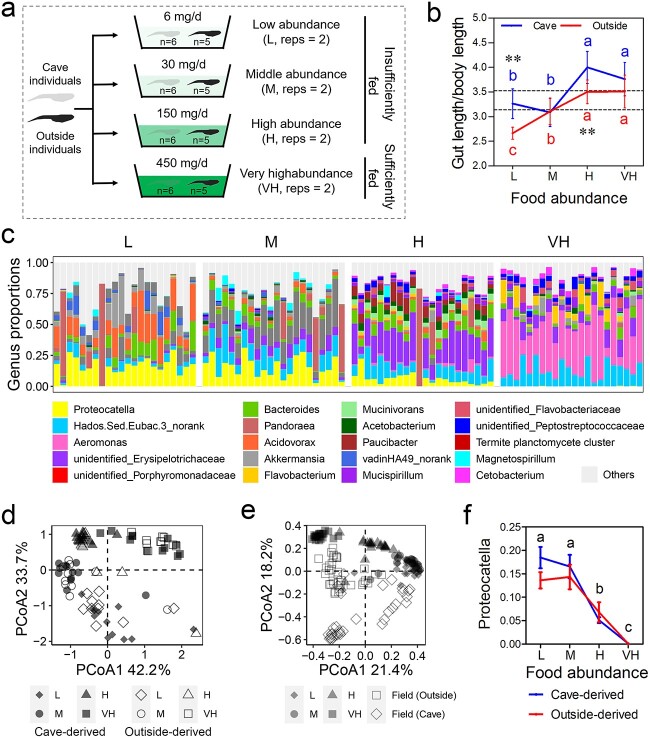
Effects of food availability on gut microbial composition of *O. rhodostigmatus* tadpoles; (A) experimental design; (B) variation in the relative gut length with food levels; different letters indicate significant differences between stages for cave- or outside-derived tadpoles (simple effects analysis for ANOVA, with significant interactive effects); asterisks indicate the differences between cave- and outside-derived individuals at each food level (simple effects analysis for ANOVA): ^*^P < .05; ^*^^*^P < .01; the values show the mean ± 95% confidence interval, and the dashed horizontal lines mark the 95% confidence interval of field-collected individuals measured before treatment; (C) microbial community structure at the genus level; (D) PCoA plot of unweighted UniFrac distances showing the similarity in microbial composition among the groups; (E) PCoA plot showing the similarity in microbial composition between laboratory and filed-collected individuals; (F) variation in the relative abundance of *Proteocatella* with food levels; the values show the mean ± SE; different letters indicate significant differences (*P* < .05, two-way ANOVA) among levels.

To verify the causal relationship between food availability and gut microbial variations, we co-cultured the cave and outside *O. rhodostigmatus* tadpoles at different food levels (L, M, H, and VH; Fig. 5A). At the end of treatment, the size of tadpoles’ storage organs (fat bodies and livers) was positively correlated with the food level and no difference was detected between cave- and outside-derived individuals (Supplementary Fig. S8 and Supplementary Table S4). However, the relative gut length of these two groups responded differently to the food availability (*P* < .05 for the interactive effect, two-way ANOVA; Fig. 5B), with significant intergroup differences under the L and H conditions (*P* < .05, simple effects). For the outside-derived individuals, this index remained similar to that of the initial values (the values of freshly collected individuals before treatment) in the M, H, and VH groups, but it decreased significantly in the L group. For the cave-derived individuals, their relative gut length did not decline in the L group and even increased in the H group.

**Figure 6:**
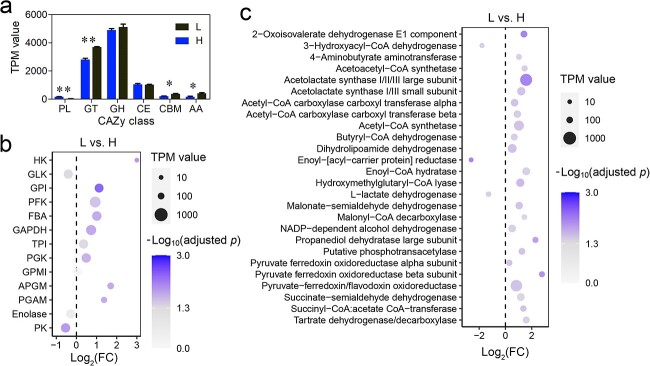
Variation in the function of gut microbiota with food levels; the gut metagenomes were compared between tadpoles from the L and H groups; (A) relative abundance of CAzymes at the class level; the values show the mean ± SE; ^*^adjusted *P* < .05; ^*^^*^adjusted *P* < .01; differences in the relative abundance of microbial glycolytic (B) and propionate- and butyrate-fermenting genes (C) between the L and H groups; AA, redox enzymes that act in conjunction with CAZymes; CBM, adhesion to carbohydrates; CE, hydrolysis of carbohydrate esters; GH, hydrolysis and/or rearrangement of glycosidic bonds; GT, formation of glycosidic bonds; PL, nonhydrolytic cleavage of glycosidic bonds.

At the end of treatment, there were no differences in the microbial alpha-diversity (Shannon index) among groups (Supplementary Fig. S9A). The microbial composition was not different between the cave- and outside-derived individuals (Fig. 5C and D and Supplementary Fig. S9B and C), but it varied with the food availability (*P* < .001, PERMANOVA) in a level-dependent manner (Supplementary Fig. S9D). The gut microbiota of the individuals from L and M groups, regardless of the tadpoles’ sources, were more similar to those of the field-collected cave individuals, while the individuals from H and VH groups were more similar to the field-collected outside individuals (Fig. 5E). We performed partial least squares regression to identify the bacterial genera that correlated with food levels in their relative abundance. This revealed that the relative abundance of *Proteocatella* increased drastically with a decrease in the food availability (Fig. 5F and Supplementary Fig. S9E and F), thereby reproducing one of the main features of cave-associated microbiota. The gut metagenomics revealed a significant difference in the functions of gut microbiota between the L and H groups (*P* < .01, PERMANOVA; Supplementary Fig. S10A). The gut metagenome of the L groups had a higher relative abundance of genes related to glycometabolism, lipid metabolism, amino acid metabolism, and organic acid metabolism than that of the H group (adjusted *P* < .05, Supplementary Fig. S10B). The gut metagenome of the L group did not show a higher relative abundance of GHs than that of the H group (Fig. 6A and Supplementary Fig. S10C), but it was richer in most of the genes related to glycolysis, propanoate metabolism, and butanoate metabolism (adjusted *P* < .05; Fig. 6B and C).

## Discussion

### The features of cave-associated gut microbiota of *O. rhodostigmatus* tadpoles

The relative abundance of *Lactobacillus*, *Cellulosilyticum*, and *Proteocatella* accounted for the primary difference in gut microbiota between cave and outside *O. rhodostigmatus* tadpoles. Lactobacilli have been used as probiotics in the breeding industry due to their ability to improve growth and prevent gastrointestinal infections [66-68]. A low-fat–high-fiber diet increases the relative abundance of *Lactobacillus* and SCFAs in animals [69], and dietary fibers accelerate the production of SCFAs mainly by stimulating intestinal *Lactobacillus*, *Bifidobacterium*, and *Akkermansia* in human [70]. In this study, a large proportion of CAZymes were assigned to *Lactobacillus* in cave individuals, suggesting the potentially important role of this genus in carbohydrate utilization. *Cellulosilyticum* bacteria were first isolated from the rumen of yaks and characterized by their numerous fibrolytic activities [71, 72]. These bacteria are obligate anaerobic, and use cellulose, cellobiose, xylan, xylose, and maltose, but not glucose, as sources of carbon and energy [73]. *Cellulosilyticum* bacteria have been identified in fish guts [74], and dietary supplementation with soluble nonstarch polysaccharides significantly increased the relative abundance of *Cellulosilyticum* in the gut of tilapia [75]. The *Lactobacillus* and particularly *Cellulosilyticum* bacteria of *O. rhodostigmatus* tadpoles seemed to be specific to the cave environment, and they disappeared in the gut of laboratory *O. rhodostigmatus* tadpoles. This partly explained why food scarcity failed to induce enrichment of glycoside hydrolases in the gut metagenome of *O. rhodostigmatus* tadpoles.

Unlike *Lactobacillus* and *Cellulosilyticum*, *Proteocatella* bacteria persistent in the guts of cave, outside, and artificially fed *O. rhodostigmatus* tadpoles. This suggests that *Proteocatella* can well adapt to changes in the diet composition and environmental conditions (e.g. pH), and thus serves as an important member in the gut of *O. rhodostigmatus* tadpoles. *Proteocatella* bacteria were first isolated from penguin guano, and can ferment acetate, propionate, and butyrate [76]. A supplement of *Proteocatella sphenisci* was reported to increase intestinal SCFA levels and improve the growth of Chu’s croaker (*Nibea coibor*) [77].

**Figure 7:**
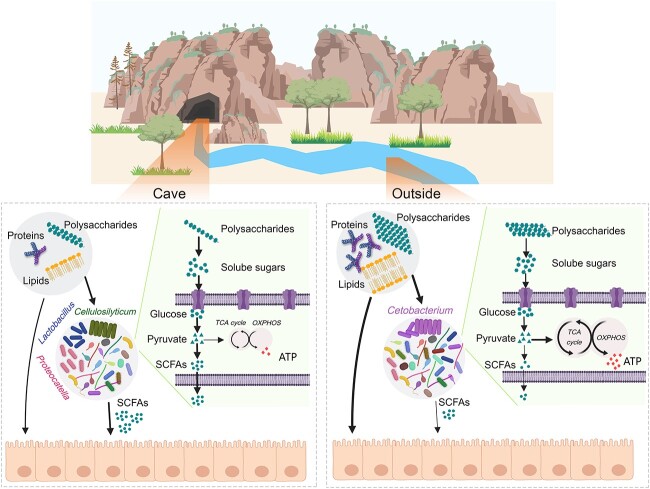
Putative mechanisms by which the gut microbiota benefit the nutrition of *O. Rhodostigmatus* tadpoles in a resource-limited environment; the thickness of the arrows denotes the relative intensity of the metabolic flows.

The high relative abundance of these three genera indicated the fiber-degrading and fermentative capacity of cave-associated microbiota. One of the most important features of the cave-associated gut metagenome was the high relative abundance of genes involved in the acquirement and degradation of resources, especially for polysaccharide hydrolysis and transport. Unlike lipids and proteins, many natural polysaccharides cannot be utilized directly by animals [78]. Enrichment in glycoside hydrolases targeting nonstarch polysaccharide has been widely reported in the gut metagenomes of animals whose major diet is rich in fiber and poor in lipid and protein. These includes ruminants [79], panda [80, 81], bamboo rats [82], and grass carp [83]. It has been suggested that the fiber-degrading gut bacteria of these animals improve their food utilization efficiency. Robust fermentation of SCFAs is another major feature of the cave-associated gut microbiota. For the gut microbiota of cave individuals, the carbon flux from carbohydrate catabolism was probably diverted into the fermentation of SCFAs rather than completed oxidation for ATP production. This metabolic pattern is in favor of the host from the perspective of resource allocation between the host and its gut microbiota. Microbiota-derived SCFAs are important metabolic substrates for the host and play physiological roles such as shaping the intestinal microenvironment and maintaining its metabolism and function [84, 85]. For example, increased microbial biosynthesis of SCFAs facilitates the adaptation of voles to stressful environments by maintaining metabolic homeostasis [39]. Collectively, the fibrolytic and fermentative gut microbiota may be beneficial to the nutrition of *O. rhodostigmatus* tadpoles (Fig. 7).

In this study, imitating food scarcity reproduced some major features (e.g. the high relative abundance of *Proteocatella* and SCFA fermenting genes) of cave-associated microbiota under laboratory conditions. These results suggest that at least some of the major features of cave-associated microbiota are likely to be related to food scarcity, a major challenge of cave environments.

### Potential associations of variations in gut microbiota with hosts’ physiology

Reduced somatic growth before metamorphic climax is a common phenomenon in amphibian tadpoles, and it means a decreased requirement for environmental resources. *O. rhodostigmatus* tadpoles experienced a development-related reduction in the growth rate and gut length after Stages 38 and 37 in cave and outside individuals, respectively. Consistent with our hypotheses, this physiological change was accompanied by a significant shift in their gut microbiota. It was interesting to find that decreased *Lactobacillus*, *Cellulosilyticum*, and *Proteocatella* accounted for the primary development-related changes in the microbial community structure. Since a higher abundance of these bacteria is potentially beneficial to the host’s nutrition, whether their decrease plays a role in the development-related reduction in host’s growth rate and gut length is an interesting question needing further investigation. Moreover, we observed different responses in the relative gut length to food scarcity between cave- and outside-derived individuals in our laboratory experiments. Maintaining a long gut is costly in terms of energy, and degeneration of the digestive tract is a common response in starving animals [86]. This was indeed the case for the outside-derived individuals, as their relative gut length decreased as the food levels declined. For the cave-derived individuals, their gut retained constant, and it even elongated under high food levels. This implies that the cave-derived individuals likely suffer less severe energy deficiencies at low food levels. Since the cave and outside tadpoles shared common genetic background, their distinct responses in the relative gut length to food scarcity were most likely due to the difference in their initial energy storage, gut microbiota, or both. According to the results of our microbial functional analyses, the distinct initial gut microbiota between the cave and outside individuals may be a plausible explanation. However, further solid evidence is still required to build a causal relationship between the gut microbiota and the resistance of *O. rhodostigmatus* tadpoles to starvation.

Whether the physiological responses of *O. rhodostigmatus* tadpoles to environmental variations may shape their gut microbiota is another interesting question. Although we observed significant changes in the gut microbiota of *O. rhodostigmatus* tadpoles with different food availability, the underlying mechanisms were unclear, especially whether the host played a role in driving the microbial changes. Regulating the oxygen level in the gut lumen is an important approach for mammals to manipulate their gut microbiota, and maintaining a low oxygen level in the gut lumen is beneficial to the host [87]. The gut microbiota of cave and outside individuals were different in their metabolic patterns. The cave gut microbiota maintained lower level of aerobic energy metabolism but a higher level of fermentation compared with their outside counterparts. This metabolic feature may be explained by the lower oxygen level in the gut of cave individuals. Therefore, further study could also focus on testing this potential “environment–host–microbe” interaction to reveal whether and how the gut microbiota play a role in the host’s adaptation to resource scarcity. For example, efforts may be put into variations in the enterocytes’ metabolic patterns and gut oxygen levels with the food availability in *O. rhodostigmatus* tadpoles.

## Conclusions

The gut microbiota of *O. rhodostigmatus* tadpoles were flexible to the host’s developmental stages and variations in environmental resources. The development-related shift in gut microbiota coincided with decreases in the growth rate and gut length of the tadpoles. The gut microbiota of cave tadpoles were distinguished by a higher relative abundance of *Cellulosilyticum*, *Lactobacillus*, and *Proteocatella* compared with the outside group. Their metagenomes were richer in fiber-degrading, carbohydrate-transporting, glycolytic, and SCFA-fermenting genes. Cave-associated microbiota showed enhanced glycolysis and production of SCFAs, but decreased aerobic metabolism and energy levels. This metabolic pattern in gut microbiota is potentially beneficial to the host’s nutrition. Laboratory studies indicated that food availability might be a driver of the gut microbial composition and function of *O. rhodostigmatus* tadpoles, and some of the major features of the cave-associated microbiota were related to resource scarcity. Collectively, the fibrolytic and fermentative gut microbiota of *O. rhodostigmatus* tadpoles may reflect their adaptation to the resource-limited environment.

## Supplementary Material

Supplementary_data_1_wrad009

Supplementary_data_2_wrad009

Supplementary_data_3_wrad009

Supplementary_data_4_wrad009

## Data Availability

Sequencing data and relevant files have been uploaded to Genome Sequence Archive (https://ngdc.cncb.ac.cn/gsub/) with the accession numbers CRA004845, CRA004802, and CRA004836.
