# Supplementary material for: Gut microbiota reflect adaptation of cave-dwelling tadpoles to resource scarcity
Source: ISME J. 2024 Jan 10;18(1):wrad009. doi: 10.1093/ismejo/wrad009 (PMC10811740; doi:10.1093/ismejo/wrad009)
Supplement: Supplementary_data_1_wrad009 [file supplementary_data_1_wrad009.docx]

**Methods and Materials**

**Identification and classification of the tadpole developmental stages**

As some similar stages cannot be easily distinguished, we classify the stages into several periods with following standard: stage 25, free-swimming tadpoles without hindlimb bud; stage 26–30, with hindlimb bud of varying length; stage 31–33, with paddle-shaped hindlimb; stage 34–36, formation of toe in hindlimb; stage 37, bending hindlimb with individual toes; stage 38, retractable hindlimb; stage 39, with long hindlimb normally in ‘N’ shape; stage 40, developed and folded hindlimb (‘W’ shape); stage 41, observation of forelimb beneath the abdomen skin; stage 42, emergence of forelimb.

**Phylogenetic analysis**

The sequence alignments of 13 mitochondrial genes (i.e., *cox1*, *cox2*, *cox3*, *cytb*, *nd1*, *nd2*, *nd3*, *nd4*, *nd4l*, *nd5*, and *nd6*) were extracted from the liver transcriptomes of 60 *O. rhodostigmatus* individuals (Cave: Outside = 24: 36, unpublished data) using MEANGS ([Song et al., 2022](#_ENREF_8)). The spurious sequences or poorly aligned regions were removed, and a 5431 bp high-quality alignment was obtained.

The partial sequences of ten nuclear genes (i.e., *evpl*, *sacs*, *disp2*, *bdnf*, *cxcr4*, *fat1*, *ntf3*, *rag1*, *ncx1*, and *sall1*) from 36 individuals (Cave: Outside = 13: 23) were obtained using PCR. These genes were commonly used for species identification and phylogenetic analysis in amphibians. The primers were designed according to the *O. rhodostigmatus* genome (unpublished data). The PCR products were sequenced and linked sequentially, followed by alignment on Mega 11.0.13 ([Tamura et al., 2021](#_ENREF_9)). Then, the linkage regions were removed manually and 5811 bp high-quality alignment was obtained.

The mitochondrial and nuclear alignments were used to construct maximum likelihood phylogenetic trees respectively using default parameters (Mega 11.0.13).

The primers for the nuclear genes

| Genes | Frimer direction | Sequence |
| --- | --- | --- |
| *bdnf* | F | 5-ACCATCCTTTTCCTTACTATGG-3 |
|  | R | 5-CTATCTTCCCCTTTTAATGGTC-3 |
| *cxcr 4* | F | 5-GGGTCACGGAAACTTTTGGC-3 |
|  | R | 5-ATTCTCCAACACGCAGTCGT-3 |
| *disp 2* | F | 5-CATCAGTTGTGCGTTTGCCA-3 |
|  | R | 5-ATGCCTGCCTTGAAGATGCT-3 |
| *evpl* | F | 5-GAAGCCCGTGGTGGAATACA-3 |
|  | R | 5-CTAATTCGTTGCGGAGTGCG-3 |
| *fat 1* | F | 5-GATGGAGGGTCACCCCAAAG-3 |
|  | R | 5-AATCGAGGCGGGTTGTCATT-3 |
| *ncx 1* | F | 5-ACAACAGTTAGGATATGGAA-3 |
|  | R | 5-CCTTCTGTTTCAATGATCAT-3 |
| *ntf 3* | F | 5-AAAGGCCACCGAGGGGAATA-3 |
|  | R | 5-ATGCACAAACGCAGGAAGTG-3 |
| *rag 1* | F | 5-TCTACACCCTGCCGTTTGTC-3 |
|  | R | 5-GGCTCTCCACGTCTCATAGC-3 |
| *sacs* | F | 5-TGTTTCGCTTTCCTTTGCGG-3 |
|  | R | 5-ATTTGGGGCACGGACTACAG-3 |
| *sall 1* | F | 5-ATTGTCAGCAGCGATCACCA-3 |
|  | R | 5-ATCAGAAGGGCTAGGAGCCA-3 |

**16S rRNA gene-based microbiome analyses (detailed version)**

For the 16S rRNA gene diversity analysis of the gut microbiota, each sample contained the whole gut content of one tadpole (Stages 25–40; see the sample sizes in Table S3). We extracted DNA from the samples using a QIAamp DNA Stool minikit (Qiagen, Valencia, CA, USA). We amplified the entire region of the 16S rRNA gene with the primers 27F (AGRGTTTGATYNTGGCTCAG) and 1492R (TASGGHTACCTTGTTASGACTT). We performed polymerase chain reaction (PCR) under the following conditions: 95 °C for 5 min, 30 cycles of 95 °C for 30 s, 50 °C for 30 s, and 72 °C for 60 s, with a final extension step at 72 °C for 7 min. We purified the PCR products with MagicPure Size Selection DNA Beads (TransGen Biotech, Beijing, China). We performed high-throughput sequencing using a PacBio platform by Mingke Biotechnology Co., Ltd. (Hangzhou, China). SMRT-Link was used to correct the original subreads to get circular consensus sequences (CCSs). The CCS were filtered using lima v1.7.0, and the primers were removed using Cutadapt 1.9.1 ([Martin, 2011](#_ENREF_6)). After removing chimera with UCHIME 8.0 ([Edgar et al., 2011](#_ENREF_5)), QIIME 2 (version 2020.6) pipeline ([Bolyen et al., 2019](#_ENREF_2)) was used to process the CCSs, and ASVs were obtained after denoising with DADA2 ([Callahan et al., 2016](#_ENREF_3)). Annotation was conducted by querying against SILVA v138 ([Quast et al., 2013](#_ENREF_7)). The absolute abundance was normalized using a standard sequence number, corresponding to the sample with the least sequences. The alpha-diversity indices and beta-diversity matrices were calculated with QIIME 2 pipeline.

For 16S rRNA gene diversity analysis of the environmental microbiota, each sample consisted of water sediment from one pool (six samples per group). We amplified the V4–V5 region with the primers 515F (GTGCCAGCMGCCGCGGTAA) and 907R (CCGTCAATTCCTTTGAGTTT). We used the following PCR thermocycling conditions: 95 °C for 5 min, 35 cycles of 95 °C for 30 s, 55 °C for 30 s, and 72 °C for 45 s, with a final extension step at 72 °C for 10 min. We performed high-throughput sequencing of amplicons using a NovaSeq 6000 System (Illumina, PE250) by Mingke Biotechnology Co., Ltd. (Hangzhou, China). The raw data were filtered using Trimmomatic 0.33 ([Bolger et al., 2014](#_ENREF_1)), and the primers were removed using Cutadapt 1.9.1 ([Martin, 2011](#_ENREF_6)). Then, the USERACH 10 was used to assemble the reads ([Edgar, 2013](#_ENREF_4)), and UCHIME 8.0 was used to remove chimera ([Edgar et al., 2011](#_ENREF_5)). QIIME 2 (version 2020.6) pipeline ([Bolyen et al., 2019](#_ENREF_2)) was used to process the sequences, and ASVs were obtained after denoising with DADA2 ([Callahan et al., 2016](#_ENREF_3)). The subsequent analysis steps are identical to those for tadpole gut microbiota.

**Chromatography (GC-MS) of SCFA measurement**

The chromatography parameters set as follows: injection volume, 1 μL; split ratio, 10:1; temperature of injectors, 250 °C; temperature of ion source: 230 °C; carrier gas (helium), 1 ml/min. The initial temperature of the oven was held stable at 90 °C, followed by a 10 °C/min increase to 120 °C, then 5 °C/ min increase to 150 °C, 25 °C/min increase to 250 °C, and stable for 2 min. We made the standard curves of acetic acid, propionic acid, isobutyric acid, butyric acid, isovaleric acid, valeric acid, and caproic acid (purchased from Sigma-Aldrich, USA) in the same manner.

**Chromatography (UPLC-MS) of metabolic profiling**

We prepared two mobile phases: 10 mM ammonium acetate in 5% acetonitrile (solvent A, pH 9) and 10 mM ammonium acetate in 95% acetonitrile (solvent B, pH 9). We set the following parameters: column temperature, 40°C; flow rate, 0.3 ml/min; injection volume, 5 μL. The mobile phase program was as follows: 0–2 min, 95% B; 2–9 min, decreasing B from 95% to 70%; 9–10 min, decreasing B from 70% to 30%; 10–11 min, 30% B; 11–11.5 min, increasing B from 30% to 95%; 11.5–15 min, 95% B. We obtained metabolite data in both positive and negative ion modes with the following settings: source temperature, 550 ℃; ion source gas 1 (GAS1), 40; ion source gas 2 (GAS2), 50; curtain gas (CUR), 35; ion spray voltage floating (ISVF), 5 500 V and −4 500 V for positive and negative ion models, respectively.

**Results**

**Supplementary figures**


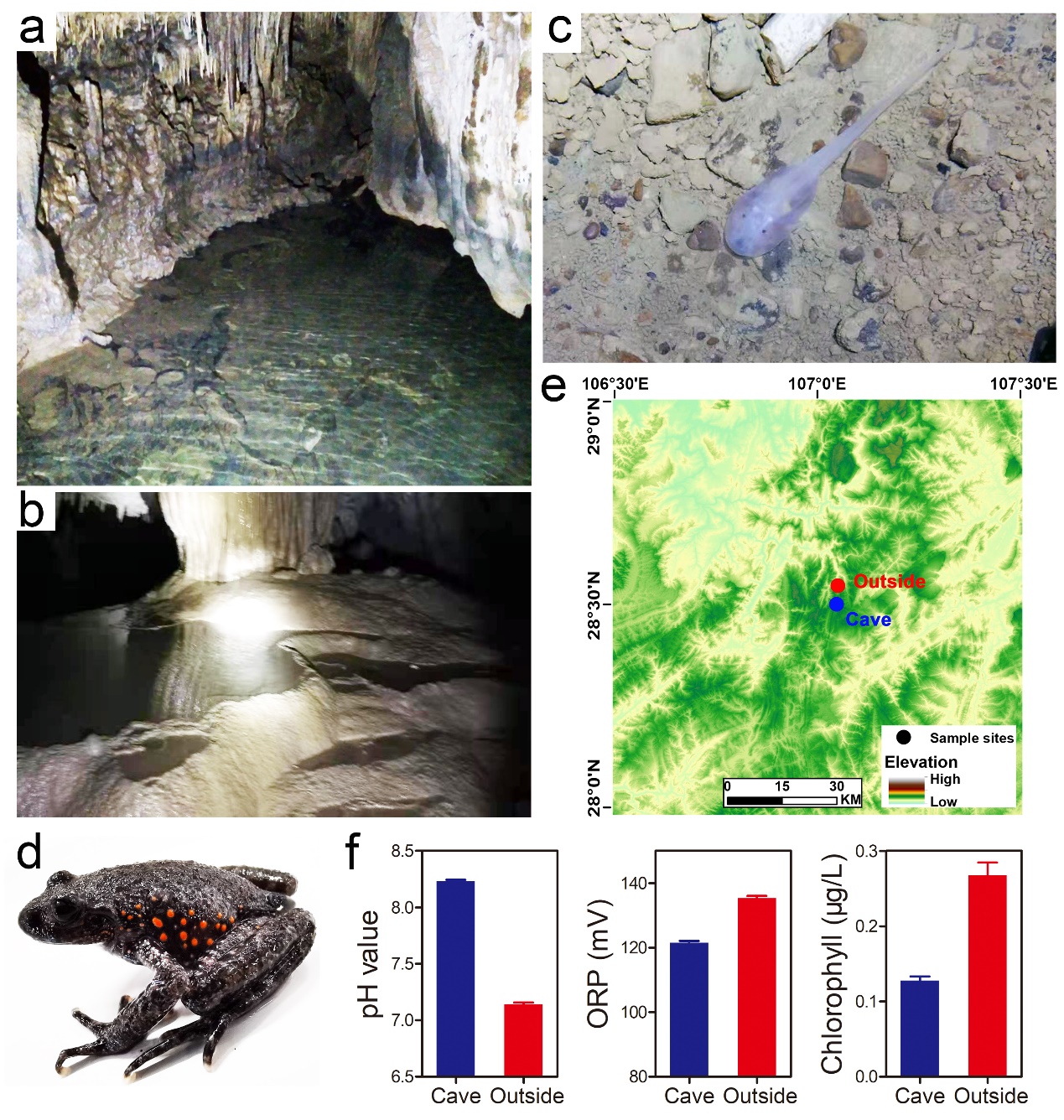


**Figure S1** Living environment and morphology of the *O. rhodostigmatus*. (a–b) Photographs presenting the natural habitat of *O. rhodostigmatus* tadpoles. (c) *O. rhodostigmatus* tadpoles in the cave. (d) A photograph presenting the morphology of juvenile *O. rhodostigmatus*. (e) Sampling sites of this study. (f) Environmental factors differing between cave and outside environments. ORP, oxidation-reduction potential. The cave individuals were collected from multiple pools in the same cave, which locate in the upstream of the outside pool. In the seasons when we collect the samples, the pools in the caves are spatially isolated with each other but connected by the running waters throughout the caves. In the flooding seasons (e.g., summer), the pools are likely fully connected. The water and sediment samples collecting for chemical and microbial analyses have covered several pools of this cave.


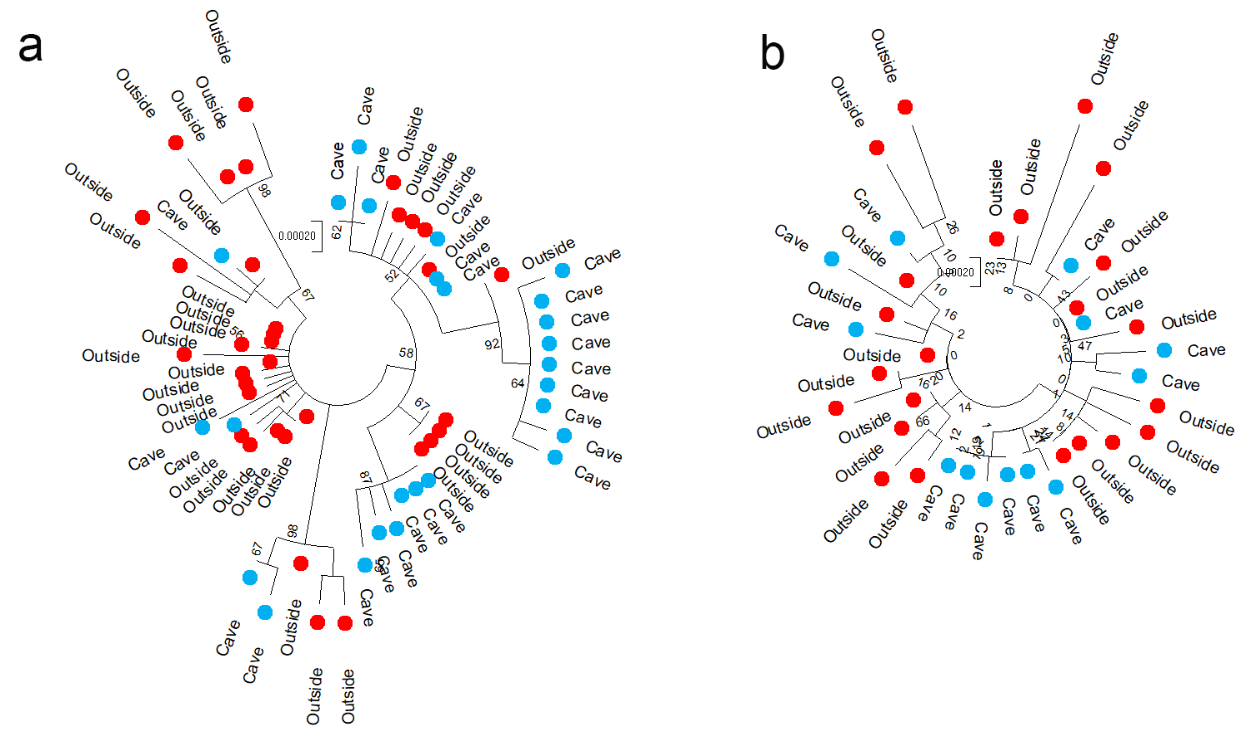


**Figure S2** Phylogenetic tree presenting the phylogenetic relationships between the cave and outside individuals. (a) Phylogenetic tree based on the sequences of 13 mitochondrial genes. (b) Phylogenetic tree based on partial sequences of ten nuclear genes. The individuals have small genetic distances with each other, and they can’t be divided into cave and outside groups genetically. Cyan, cave individuals; red, outside individuals.


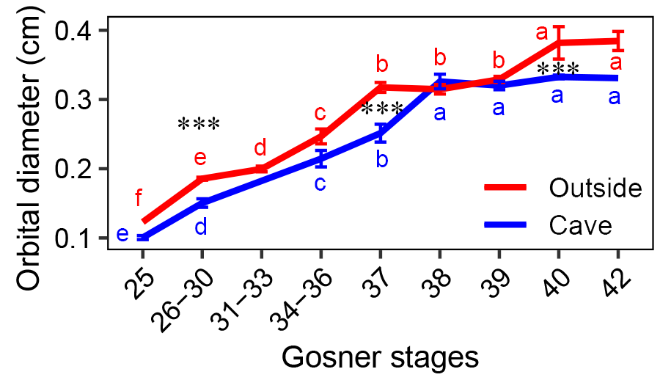


**Figure S3** Variations of orbital diameter with environments and developmental stages. Blue and red letters denote significant differences between stages for cave and outside individuals, respectively, and asterisks denote differences between cave and outside individuals at given stages (simple effects analysis for ANOVA, with significant interactive effect): * *p* < 0.05; ** *p* < 0.01; *** *p* < 0.001.


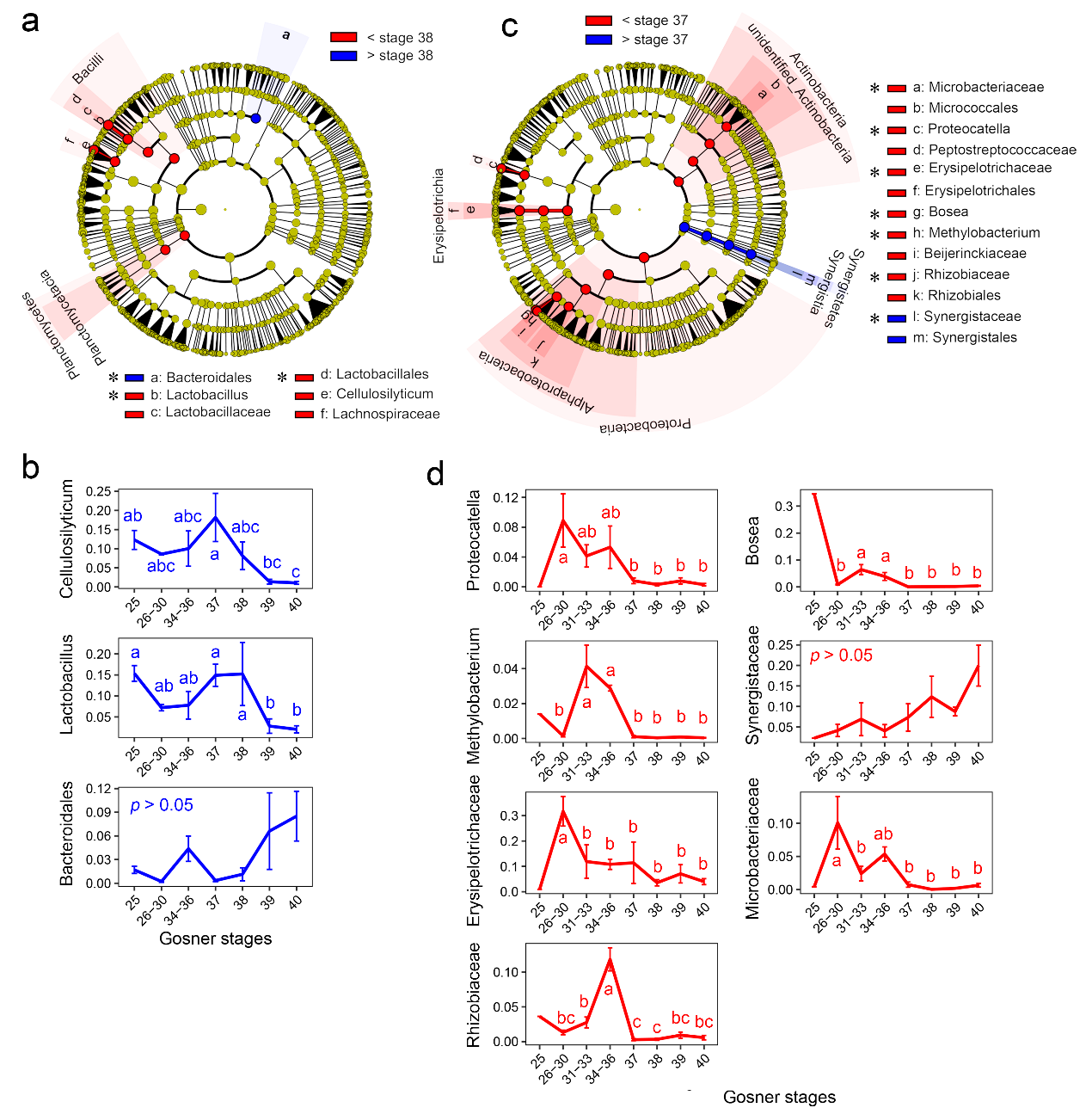


**Figure S4** Variations in the gut microbiota of *O. rhodostigmatus* tadpoles with development. (a) Microbial differential analyses for cave individuals based on LEfSe at threshold of *p* < 0.01 (Kruskal-Wallis and Wilcoxon tests) and linear discrimination analysis score (LDA) > 2. Asterisks denote most significant differential taxa (LDA > 4). (b) The variation in relative abundance of differential bacteria (based on LEfSe) with developmental stages in cave tadpoles. The y axis denotes the proportions. The data were analyzed with one-way ANOVA and LSD post-hoc test. Different letters indicate significant difference between groups at threshold of *p* < 0.05. (c) Microbial differential analyses for outside individuals based on LEfSe at threshold of *p* < 0.01 (Kruskal-Wallis and Wilcoxon tests) and linear discrimination analysis score (LDA) > 2. Asterisks denote most significant differential taxa (LDA > 4). (d) The variation in relative abundance of differential bacteria (based on LEfSe) with developmental stages in outside tadpoles. The y axis denotes the proportions. The data were analyzed with one-way ANOVA and LSD post-hoc test. Different letters indicate significant difference between groups at threshold of *p* < 0.05.


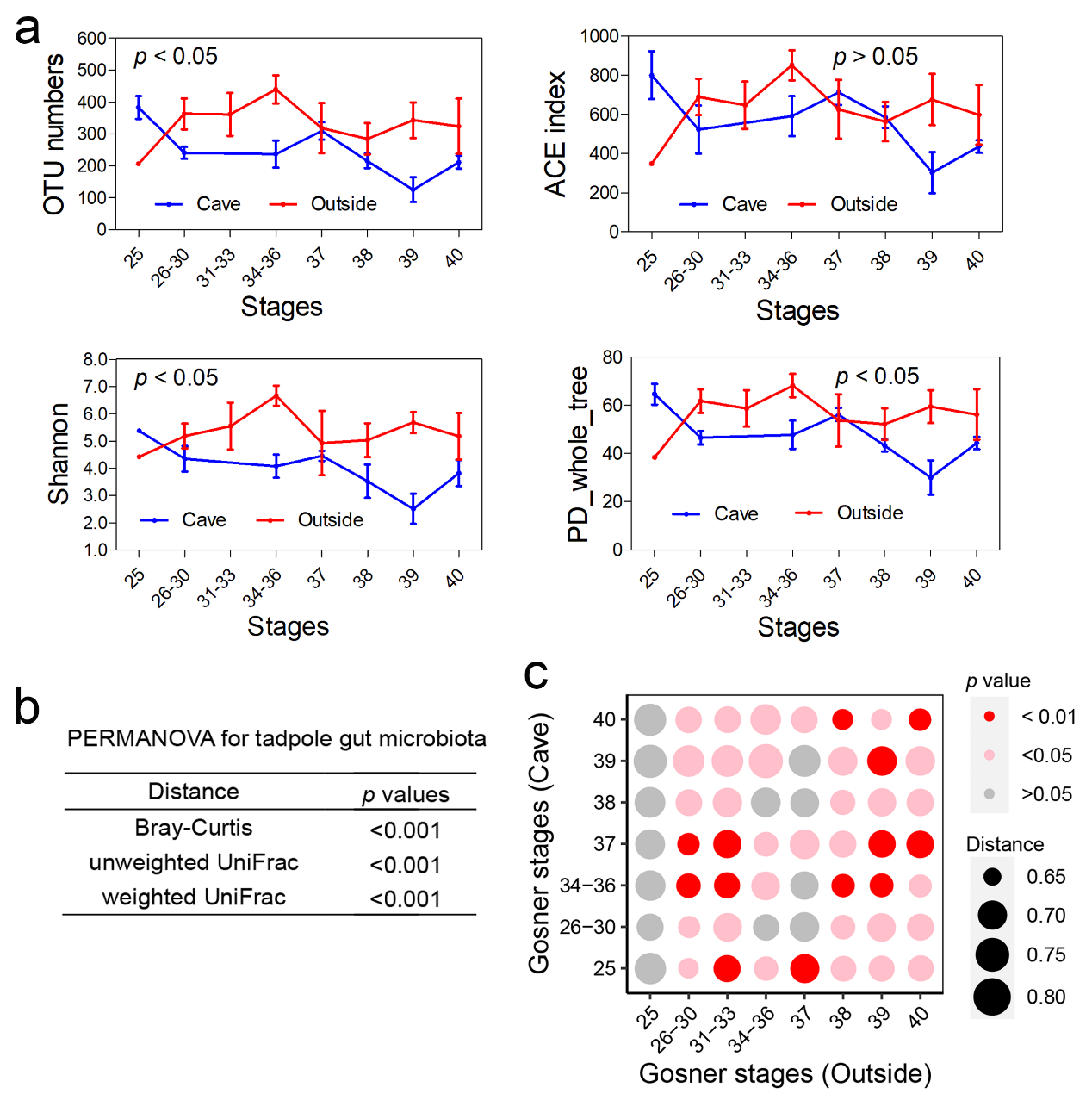


**Figure S5** Additional information on community structure of gut microbiota. (a) Alpha diversity. The data are analyzed by two-way ANOVA. There are no significant differences in alpha diversity between developmental stages. The gut microbiota of cave and outside individuals differ in OTU numbers, Shannon, and PD_whole_tree indices, but not in ACE index (denoted by *p* values). (b) Differences in bacterial beta-diversity between cave and outside individuals. The influences of developmental stages are not considered. (c) Pairwise PERMANOVA on microbial beta-diversity between cave and outside individuals at different stages (unweighted UniFrac distance).


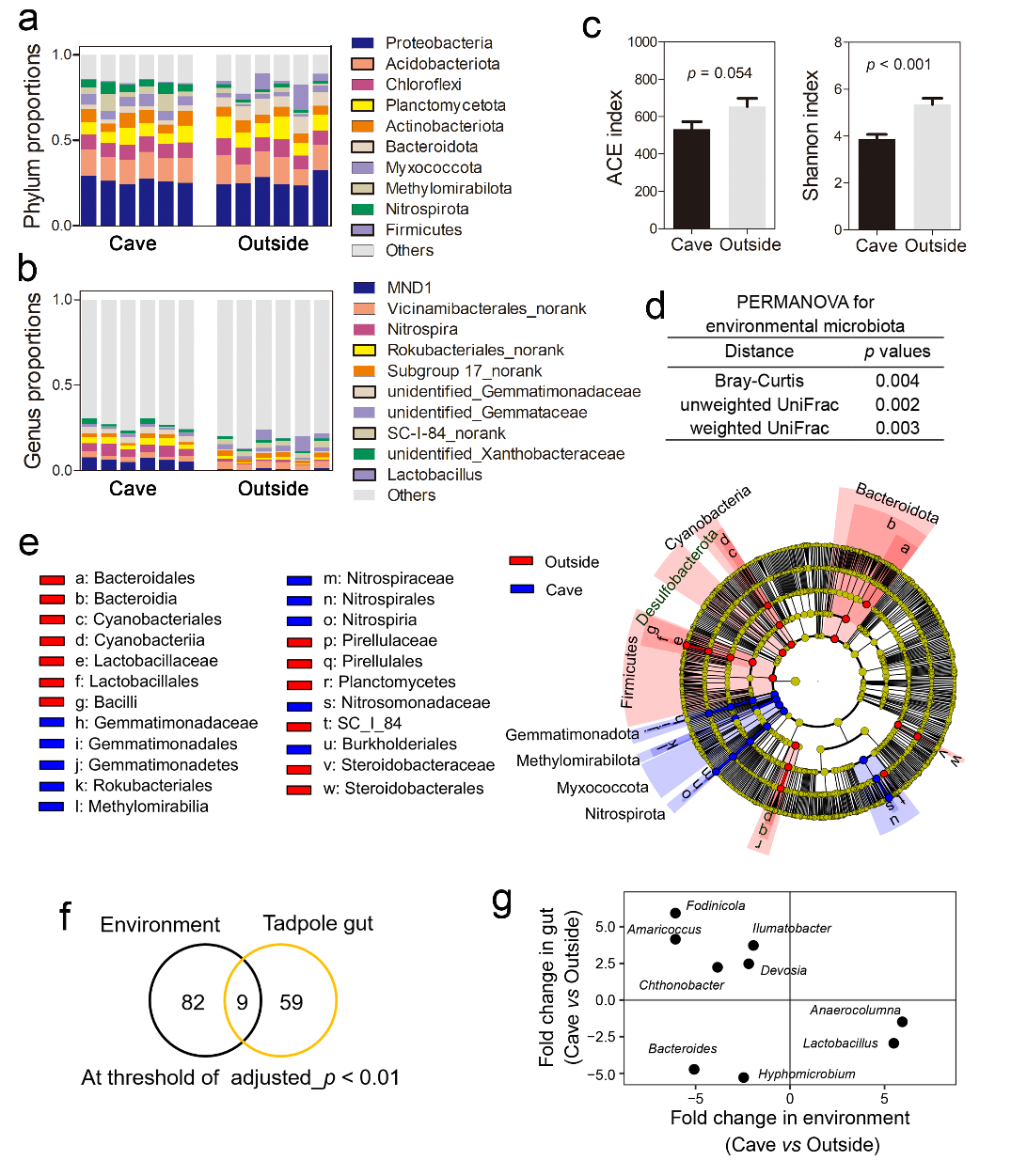


**Figure S6** Comparative analyses on cave and environmental microbiota of cave and outside environments. (a–b) Community structure at phylum (a) and genus (b) levels. (c) Differences in bacterial alpha diversity indices. The *p* values present the results of Mann-Whitney U test. (d) Table presenting the results of PERMANOVA on the bacterial beta-diversity indices. (e) Results of LEfSe (performed on Galaxy platform, http://huttenhower.sph.harvard.edu/galaxy/). Significant threshold is set at *p* < 0.05 for Kruskal-Wallis test and Wilcoxon test, and LDA (linear discriminant analysis) score > 2 for discriminative features. (f) Venn diagram presenting the differential bacterial genus between cave and outside groups for environmental (black) and gut (orange) microbiotas. Nine differential genera were differed between cave and outside groups for both environment and tadpole gut. The threshold of differential genus was adjusted_*p* < 0.05 (Mann-Whitney U test and BH correction). (g) Point plot presenting the fold change (cave *vs* outside) in relative abundance of the nine shared genera. The x and y axes were the fold changes in the environment and tadpole gut, respectively. Thus, points located in the 2^nd^ and 4^th^ quadrants have opposite variation trends in the environment and tadpole gut.


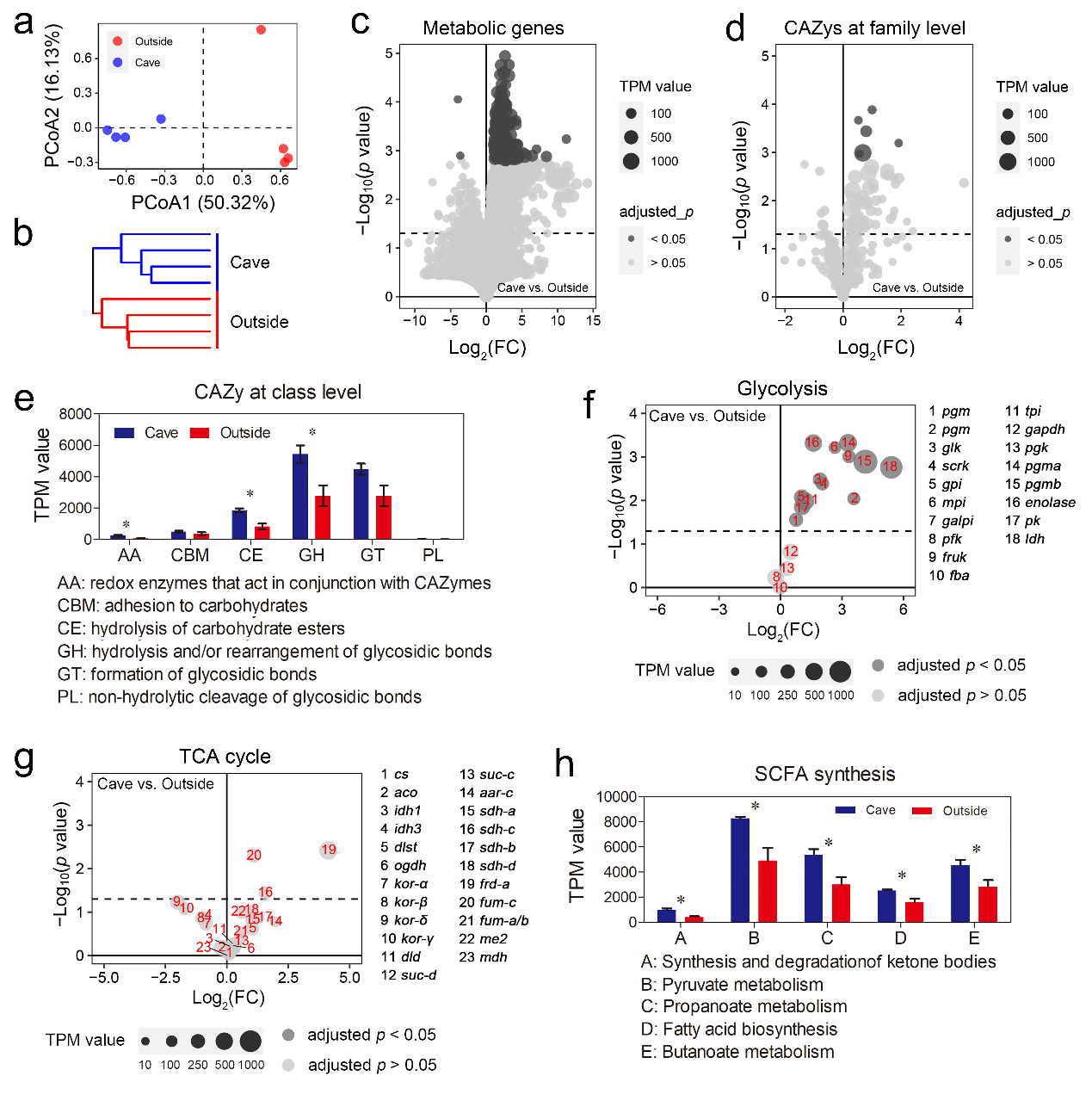


**Figure S7** Metagenomic analyses on the tadpole gut microbiota. (a) PCoA scatter showing the differences in gut metagenome between cave and outside individuals. (b) Results of Hcluster analysis on metagenomic data. (c) Volcano plot presenting the variations in relative abundance of KEGG-annotated metabolic genes (Students’ *t* test and BH correction). FC, fold change. The size of the dots denotes the maximum TPM value across samples. (d) Volcano plot presenting the variations in relative abundance of carbohydrate-active enzymes (CAZys) at family level (Students’ *t* test and BH correction). (e) Relative abundance of CAZys at class level (mean ± se). (f–g) Volcano plots presenting the variation in relative abundance of genes involved in glycolysis (f) and sugar PTS components (g). The horizontal axis denotes the fold change (FC) in TPM values, and the vertical axis stands for *p* values. The size of the dots denotes the maximum TPM value across samples. (h) Relative abundance of metabolic pathways related to SCFA synthesis (mean ± se). *, adjusted_*p* < 0.05 (Students’ *t* test and BH correction).


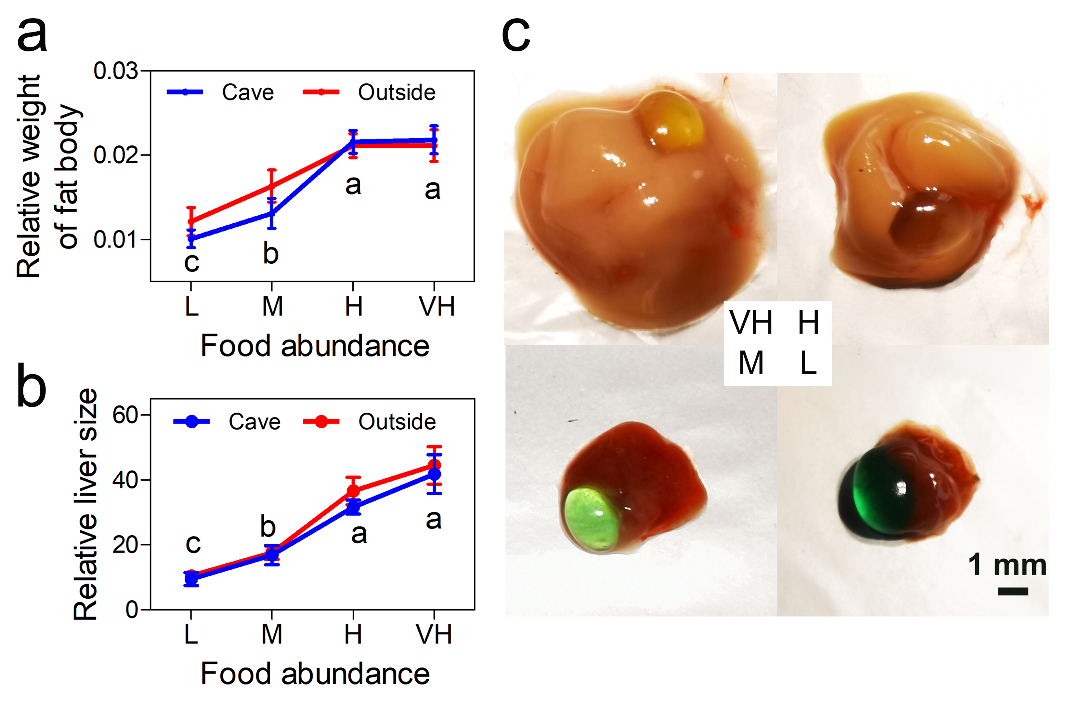


**Figure S8** Influences of food level on tadpole nutrient storage. For each food level, the data were collected for ten outside-derived and twelve cave-derived individuals. (a) Variations in the relative weight of fat body (ratio of fat body weight to body weight) between groups. Different letters denote significant difference between food levels (*p* < 0.05, two-way ANOVA followed by S-N-K post-hoc test). (b) Variations in the relative liver size between groups. The relative liver size of each individual was quantified with Image-Pro Plus. Different letters denote significant difference between food levels (*p* < 0.05, ANCOVA followed by S-N-K post-hoc test, body weight as the covariate). (c) Typical morphology of the liver and gall bladder at different food levels. The liver can be a major fat depot in tadpoles when food is sufficient, and livers with high fat levels have an oil appearance (e.g., the H and VH group) ([Zhu et al., 2019](#_ENREF_11)). The gall bladders of tadpoles from L group are enlarged with a dark green color, which are sign of starvation in tadpoles ([Zhu et al., 2020](#_ENREF_10)). It suggests our food levels are reasonable.


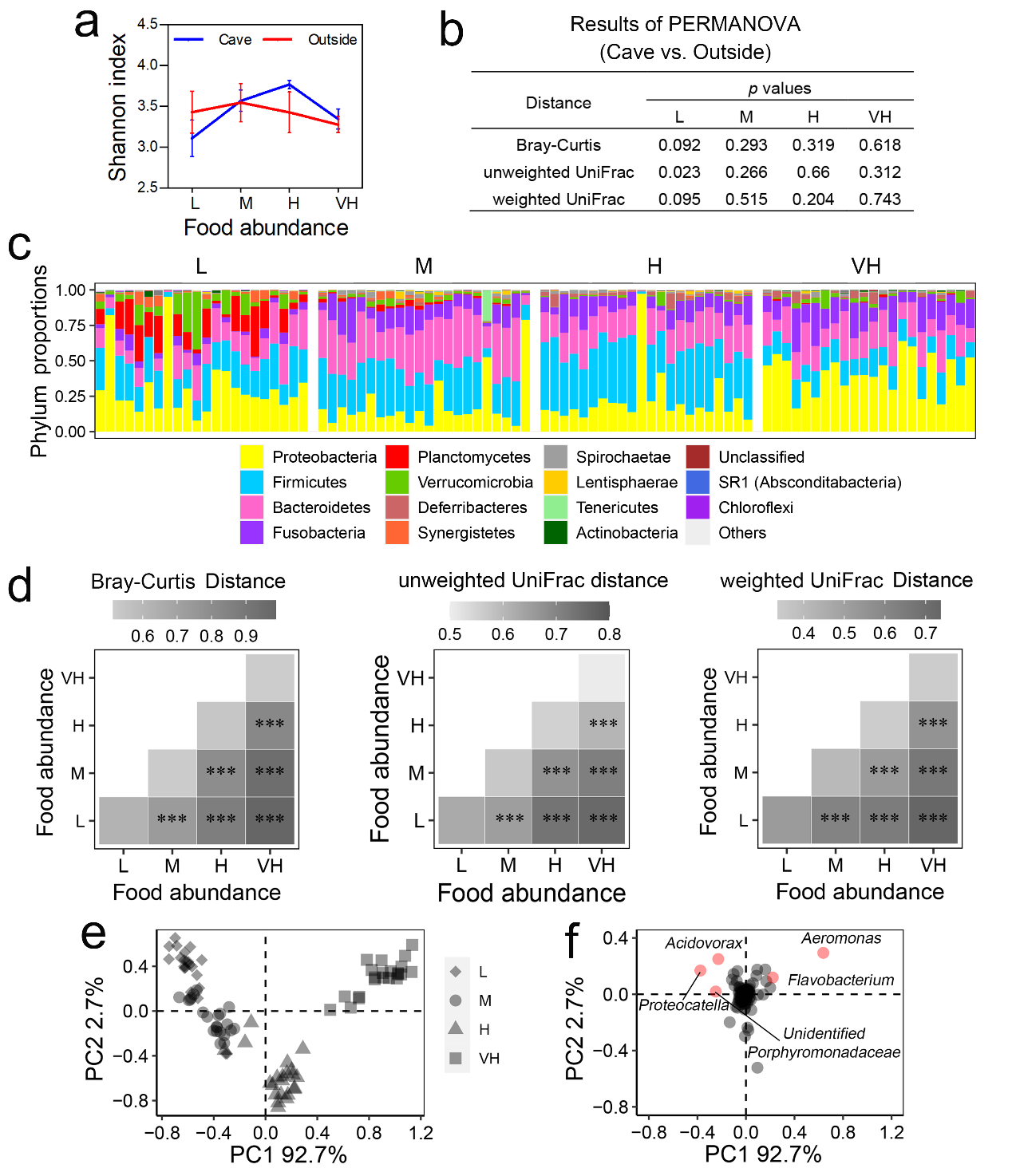


**Figure S9** Variations of tadpole gut microbiota with food levels. (a) Variation in the gut microbial alpha-diversity with food levels. The values show the mean ± SE. (b) Results of PERMANOVA on beta-diversity of gut microbiota between cave and outside individuals at given food levels. (c) Bacterial compositions at phylum level. (d) Heatmap presenting the average distances in gut microbiota between tadpole from different food levels, regardless of their source from cave and outside environments. Asterisks denote significant differences in beta-diversity between food levels (pairwise PERMANOVA). ***, *p* < 0.001. (e–f) Scatter (e) and loading (f) plots of partial least squares regression analysis. Food level and microbial abundance matrix are set as y and x values, respectively, in this model. The microbial genera with significant contribution to the model are highlighted in red color.


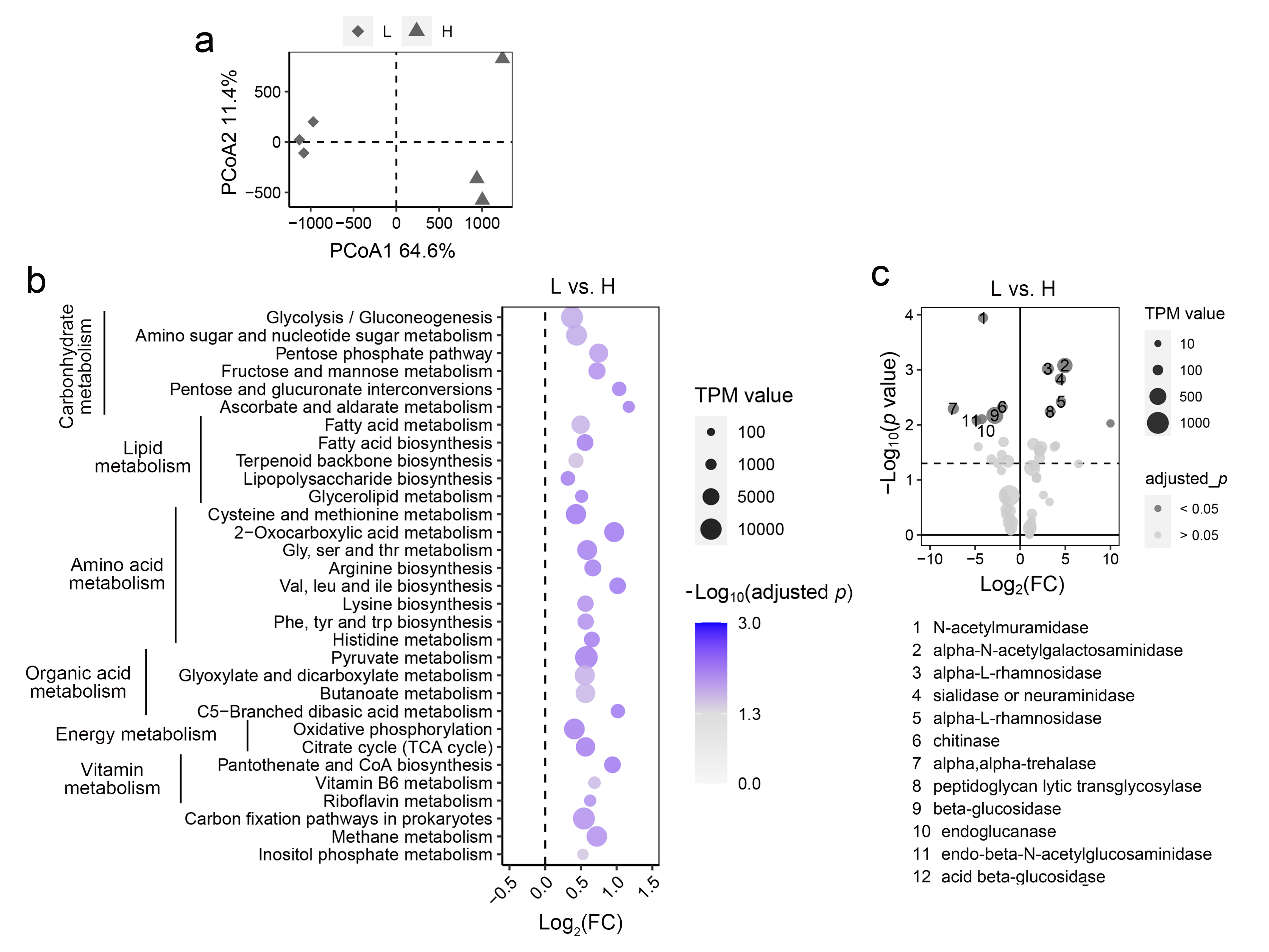


**Figure S10** Functional variations in gut microbiota with food level. (a) PCoA plot showing the similarity in gut metagenome between samples. (b) Differential KEGG metabolic pathways between L and H gut metagenomes. Only the items meeting adjusted *p* < 0.05 are presented. (c) Volcano plot presenting the differences in the relative abundance of glycosylhydrolases between L and H groups. The size of the dots denotes the maximum TPM value across samples.

**References**

Bolger, A.M., Lohse, M., Usadel, B., 2014. Trimmomatic: a flexible trimmer for Illumina sequence data. Bioinformatics 30, 2114-2120.

Bolyen, E., Rideout, J.R., Dillon, M.R., Bokulich, N.A., Abnet, C.C., Al-Ghalith, G.A., Alexander, H., Alm, E.J., Arumugam, M., Asnicar, F., Bai, Y., Bisanz, J.E., Bittinger, K., Brejnrod, A., Brislawn, C.J., Brown, C.T., Callahan, B.J., Caraballo-Rodríguez, A.M., Chase, J., Cope, E.K., Da Silva, R., Diener, C., Dorrestein, P.C., Douglas, G.M., Durall, D.M., Duvallet, C., Edwardson, C.F., Ernst, M., Estaki, M., Fouquier, J., Gauglitz, J.M., Gibbons, S.M., Gibson, D.L., Gonzalez, A., Gorlick, K., Guo, J., Hillmann, B., Holmes, S., Holste, H., Huttenhower, C., Huttley, G.A., Janssen, S., Jarmusch, A.K., Jiang, L., Kaehler, B.D., Kang, K.B., Keefe, C.R., Keim, P., Kelley, S.T., Knights, D., Koester, I., Kosciolek, T., Kreps, J., Langille, M.G.I., Lee, J., Ley, R., Liu, Y.-X., Loftfield, E., Lozupone, C., Maher, M., Marotz, C., Martin, B.D., McDonald, D., McIver, L.J., Melnik, A.V., Metcalf, J.L., Morgan, S.C., Morton, J.T., Naimey, A.T., Navas-Molina, J.A., Nothias, L.F., Orchanian, S.B., Pearson, T., Peoples, S.L., Petras, D., Preuss, M.L., Pruesse, E., Rasmussen, L.B., Rivers, A., Robeson, M.S., Rosenthal, P., Segata, N., Shaffer, M., Shiffer, A., Sinha, R., Song, S.J., Spear, J.R., Swafford, A.D., Thompson, L.R., Torres, P.J., Trinh, P., Tripathi, A., Turnbaugh, P.J., Ul-Hasan, S., van der Hooft, J.J.J., Vargas, F., Vázquez-Baeza, Y., Vogtmann, E., von Hippel, M., Walters, W., Wan, Y., Wang, M., Warren, J., Weber, K.C., Williamson, C.H.D., Willis, A.D., Xu, Z.Z., Zaneveld, J.R., Zhang, Y., Zhu, Q., Knight, R., Caporaso, J.G., 2019. Reproducible, interactive, scalable and extensible microbiome data science using QIIME 2. Nat Biotechnol 37, 852-857. https://doi.org/10.1038/s41587-019-0209-9

Callahan, B.J., McMurdie, P.J., Rosen, M.J., Han, A.W., Johnson, A.J.A., Holmes, S.P., 2016. DADA2: High-resolution sample inference from Illumina amplicon data. Nat Methods 13, 581-583. https://doi.org/10.1038/nmeth.3869

Edgar, R.C., 2013. UPARSE: highly accurate OTU sequences from microbial amplicon reads. Nat. Methods 10, 996-998. https://doi.org/10.1038/nmeth.2604

Edgar, R.C., Haas, B.J., Clemente, J.C., Quince, C., Knight, R., 2011. UCHIME improves sensitivity and speed of chimera detection. Bioinformatics 27, 2194-2200. https://doi.org/10.1093/bioinformatics/btr381

Martin, M., 2011. CUTADAPT removes adapter sequences from high-throughput sequencing reads. EMBnet J. 17, 10-12. https://doi.org/10.14806/ej.17.1.200

Quast, C., Pruesse, E., Yilmaz, P., Gerken, J., Schweer, T., Yarza, P., Peplies, J., Glöckner, F.O., 2013. The SILVA ribosomal RNA gene database project: improved data processing and web-based tools. Nucleic Acids Res. 41, D590-D596. https://doi.org/10.1093/nar/gks1219

Song, M.-H., Yan, C., Li, J.-T., 2022. MEANGS: an efficient seed-free tool for de novo assembling animal mitochondrial genome using whole genome NGS data. Briefings in Bioinformatics 23, bbab538. https://doi.org/10.1093/bib/bbab538

Tamura, K., Stecher, G., Kumar, S., 2021. MEGA11: Molecular Evolutionary Genetics Analysis version 11. Molecular Biology and Evolution 38, 3022-3027.

Zhu, W., Chang, L., Zhao, T., Wang, B., Jiang, J., 2020. Remarkable metabolic reorganization and altered metabolic requirements in frog metamorphic climax. Front Zool 17, 30. https://doi.org/10.1186/s12983-020-00378-6

Zhu, W., Zhang, M., Chang, L., Zhu, W., Li, C., Xie, F., Zhang, H., Zhao, T., Jiang, J., 2019. Characterizing the composition, metabolism and physiological functions of the fatty liver in *Rana omeimontis* tadpoles. Frontiers in Zoology 16, 42. https://doi.org/10.1186/s12983-019-0341-x
